# Genetic enhancement of root, tuber and cereal crops via pangenomics, multi-omics integration and AI-driven prediction

**DOI:** 10.3389/fpls.2026.1793924

**Published:** 2026-05-13

**Authors:** Simbo Diakite, Prince E. Norman, Lansana Kamara, Necla Pehlivan, Meisam Zargar

**Affiliations:** 1 Department of Agrobiotechnology, Institute of Agriculture, Peoples Friendship University of Russia (RUDN) University, Moscow, Russia; 2 Germplasm Improvement and Seeds System, Sierra Leone Agricultural Research Institute (SLARI), Freetown, Sierra Leone; 3 Faculty of Art and Sciences, Department of Biology, Recep Tayyip Erdogan University, Rize, Türkiye; 4 Professor of plant protection, Department of Agrobiotechnology, Agrarian Technological Institute, RUDN University, Moscow, Russia

**Keywords:** artificial intelligence, crops, environmental adaptation, genetic enhancement, multi-omics, pangenomics, personalized breeding, precision agriculture

## Abstract

Breeding root, tuber, and cereal crops faces the critical challenge of unlocking extensive genetic variation and addressing complex gene-environment interplays to boost yield, quality, and resilience. Recent technological advances in pangenomics, multi-omics data integration, and artificial intelligence (AI)-driven predictive modeling offer unparalleled opportunities to transform crop improvement. Pangenomics transcends the limitations of single reference genomes by encompassing the full genomic diversity within species, capturing critical structural variations and rare alleles that underpin stress tolerance and productivity traits. When layered with multi-omics datasets spanning genomics, transcriptomics, proteomics, and metabolomics, a holistic insight is gained into molecular networks governing plant adaptation and development. State-of-the-art AI methodologies harness these complex datasets, enabling precise genomic selection, accurate trait prediction, and discovery of novel candidate genes, thereby optimizing breeding pipelines. This review presents current knowledge on how this synergistic approach heralds a new era of climate-smart agriculture, empowering resilient, high-performing cultivars essential for global food security amid escalating environmental uncertainties with a particular focus on root, tuber and cereal crop genetic enhancement through pangenomics and multi-omics integration and AI-driven predictive modeling. Together, these innovations enable tailored breeding strategies that align genetic potential with environmental specificity and farmer needs, while highlighting the remaining hurdles-data standards, model interpretability, computational cost, and equitable access-that must be addressed to realize widespread impact. Demonstrated in staple crops such as maize, rice, wheat, potato, and cassava, this integrated framework accelerates genetic gain by reducing breeding cycles and facilitating allele introgression from wild relatives. The integrative approach also provides a better understanding of resolving persistent hurdles around data standardization, interpretability, computational demands, and equitable technology access. We recommend, (i) training on diverse, field-collected datasets; (ii) integrating envirotyping covariates into genomic selection to quantify G×E interactions; (iii) adopting standardized metadata schemas; and (iv) fostering interdisciplinary collaboration.

## 1 Introduction

Root, tuber, and cereal crops form the backbone of food security and nutrition for billions of people worldwide. However, the sustainable improvement of these staple crops remains an urgent global challenge due to the need to harness extensive genetic diversity and manage multifaceted gene–environment interactions in an era of environmental volatility. Breeders routinely face the demands of achieving higher yields, better quality, and greater resilience, all while combating major abiotic stresses such as prolonged droughts, salinization, and temperature extremes. These obstacles are compounded by the escalating threat of biotic pressures, including persistent and emerging pests and pathogens, which evolve in response to shifting agricultural practices and climate change (Uga, 2021; Xiong et al., 2022; Singh et al., 2023; Beyene et al., 2024; Pfordt and Paulus, 2025; Conde et al., 2025). Despite progress in conventional and molecular breeding strategies, progress is still limited by the unpredictable nature of genotype environment interactions, long breeding cycles, and the ongoing challenge of accurately phenotyping diverse germplasm collections (Yang et al., 2024; Andrade-Piedra et al., 2025).

The emergence of high-throughput “omics” technologies has positioned plant science on the cusp of a paradigm shift. In recent years, pangenomics, a discipline entailing the creation and comparative analysis of entire collections of genomes within a species or crop group has provided unprecedented insight into the full spectrum of genetic variation underlying root, tuber, and cereal crops (Li et al., 2022; Liu et al., 2025a; Jayakodi et al., 2021). Unlike traditional reference genomes, pangenomes unveil structural variants, accessory genes, and presence-absence variation, enabling the identification of alleles linked to key agronomic traits or environmental adaptation that might otherwise be overlooked. Multi-omics integration takes this further by combining genomics, transcriptomics, proteomics, metabolomics, and phenomics, painting a multidimensional map of plant biology and its interaction with the environment (Yang et al., 2021; Zhang et al., 2022; Kumar et al., 2023). These advances allow researchers to better parse the layers of regulation and interaction that define complex traits, offering a holistic strategy to accelerate variety improvement in crop breeding programs.

Concurrently, artificial intelligence (AI) is transforming the landscape of plant breeding. Through the adoption of machine learning and deep learning methods, AI systems can analyze and integrate the massive, high-dimensional datasets produced by pangenomics and multi-omics research (Pai et al., 2025; Mishra et al., 2022). Such AI-driven predictive models are capable of uncovering intricate relationships among genotype, phenotype, management, and environment, giving rise to more accurate genomic selection and trait prediction tools (Farooq et al., 2024; Novielli et al., 2024). More than just accelerating breeding cycles, these techniques can identify candidate genes and regulatory networks for editing and introgression, thus streamlining decision-making for breeders and advancing resource-efficient, targeted breeding pipelines. AI’s capacity to generate explainable predictions is increasingly enabling cross-crop learning, dynamic modeling of future performance, and proactive management of cultivation risk (Bose et al., 2024). For instance, Paudel et al. (2023) demonstrated how neural networks independently identify essential features, enabling accurate crop yield predictions. Moreover, a new hybrid Long Short-Term Memory + XGBoost (LSTM-XGB) model uses SHapley Additive Explanations (SHAP) for yield estimates via feature contribution analysis. Osibo et al. (2023) introduced a hybrid LSTM-XGB model for wheat yield prediction that outperformed State-of-the-Art (SOTA) models. The SHAP tool analysis clarified LSTM feature extraction and XGB training, underscoring feature contribution to yield predictions and improving model interpretability and application in agriculture. Deep Learning (DL) has contributed in transforming crop classification (Kamrul et al., 2000), fruit grading (Hossain et al., 2018), pest detection (Türkoglu and Hanbay, 2019), plant disease identification (Trivedi et al., 2021), and weed discernment (Mishra et al, 2022). Wei et al. (2022) assessed the internal interpretability of DL models for fruit leaf disease prediction using an advanced ResNet-Attention model with the attention mechanism. Ribeiro et al. (2016) and Selvaraju et al. (2017) found that model feature extraction performance using SmoothGrad, Local Interpretable Model-agnostic Explanations (LIME) and GradCAM interpretative algorithms differs across agricultural categorization tasks. Nahiduzzaman et al. (2023) also introduced an Explainable Artificial Intelligence (XAI) framework that uses the parallel depth-wise conventional neutral network (PDS-CNN) model and SHAP for XAI to analyze specific image sections for mulberry leaf disease classification, improving categorization. Mehedi et al., 2022) used a transfer learning strategy with the EfficientNetV2L model and XAI, specifically LIME, to make sure that finding plant diseases was reliable and transparent. Khalid and Karan (2024) showed that transfer learning improves model efficacy by using pre-existing reliable data and integrating XAI via GradCAM to visualize disease signals in plant imagery. Gilbert et al. (2023) developed XAI models for Black Sigatoka detection in banana plants using LIME and Integrated Gradients to create transparent and interpretable models, demonstrating the dynamism of agricultural innovation and sophisticated machine learning.

Despite the remarkable promise of combining pangenomics, multi-omics, and AI, several barriers persist such as data standards, model interpretability, computational cost, and equitable access. Data harmonization is nontrivial, as raw datasets are generated with diverse protocols, from disparate sources, and across different environments and timeframes. The lack of standardized data standards such as high dimensionality, heterogeneity, and frequency of missing values across data types limits the integration of pangenomics, multi-omics, and AI (Baião et al., 2025). Interpretability of AI-driven models—often referred to as the “black box” problem—remains a stumbling block, as researchers must ensure that models not only deliver accurate predictions but also yield actionable biological insights (Liu et al., 2025a; Jayakodi et al., 2021). The integration of pangenomics, multi-omics, and AI presents computational cost barriers. The traditional CPU-workflows take many hours or days to process a single genome, thereby limiting the study of large populations or reanalysis of data when new tools become available. However, the advent of Embarrassingly_FASTA, a GPU-accelerated preprocessing pipeline, has revolutionized this landscape. This technique enables the retention of raw FASTQ data and the use of highly discounted ephemeral cloud infrastructure, reducing compute spend from approximately $100 per genome to < $1 per genome. This reduction in cost makes it feasible to keep the original DNA data and reprocess it whenever new tools become, thus enabling recomputable, population-scale pangenomics and next-generation genomic models (Walsh and Njie, 2026). To overcome these challenges, scientists utilize computational methods that leverage statistical and machine learning techniques to uncover complex biological patterns and improve our understanding of disease mechanisms. Validation and benchmarking across species, environments, and agronomic systems are also essential for moving from proof-of-concept findings to applied, real-world impact (Zhang et al., 2022).

Nonetheless, the integration of these technologies represents a transformative shift for root, tuber, and cereal crop breeding. By bridging the gaps between diverse “omics” disciplines and AI-powered analytics, breeding programs can more effectively unlock hidden genetic potential, decipher genotype–environment interactions, and breed for much-needed resilience and productivity. This comprehensive, data-driven approach not only accelerates genetic gain but also paves the way for food systems that can withstand future environmental and socioeconomic challenges (Li et al., 2022; Liu and Cui, 2025).

This review synthesizes key advances and current developments at the intersection of pangenomics, multi-omics integration, and AI, focusing on their collective potential to enable more resilient, productive root, tuber and cereal crop varieties and drive the next generation of breeding technologies.

## 2 Review methodology

### 2.1 Literature search strategy

A systematic search of Web of Science, Scopus, Springer Nature, PubMed/PMC, ScienceDirect, and MDPI was carried out, focusing on selected root, tuber and cereal crops in relation to genetic enhancement through pangenomics and multi-omics integration and AI-driven predictive modeling. The search employed the Boolean combinations of keywords including rice, maize, wheat, barley, potato, cassava and yam, multi-omics integration and AI-driven predictive modeling to ensure broad and inclusive coverage. Moreover, the references of selected articles or papers were reviewed to identify supplementary studies pertinent to study.

### 2.2 Inclusion and exclusion criteria

Inclusion criteria were peer-reviewed articles, reviews, and field experiments reporting on rice, maize, wheat, barley, potato, cassava and yam genetic enhancement through pangenomics and multi-omics integration and AI-driven predictive modeling. Representative high-impact and recent sources were prioritized in the synthesis. Both review and experimental studies with qualitative or quantitative data were accepted, with priority given to research addressing genetic enhancement of the selected root, tuber and cereal crops through pangenomics and multi-omics integration and AI-driven predictive modeling. Non-peer-reviewed works, studies that lacked clear methodologies, and those unrelated to the subject were excluded.

### 2.3 Time period covered and quality assessment

The review focused on recent literature, with approximately 89% of studies published between 2020 and 2025, capturing current advances in genetic enhancement of selected root, tuber and cereal crops through pangenomics and multi-omics integration and AI-driven predictive modeling. The remaining 11% of studies predated 2020, providing essential background and historical context. This approach ensures that the review reflects up-to-date scientific insights, emerging technologies, and evolving management practices while acknowledging long-term trends in rice, maize, wheat, barley, potato, cassava and yam research.

The selected articles were assessed using a structured framework emphasizing methodological relevance. Peer-reviewed publications from reputable sources were retained after removing duplicates. Each study was reviewed for relevance to rice, maize, wheat, barley, potato, cassava and yam. ensuring that the review provides scientifically credible evidence supporting sustainable genetic enhancement of these crops through pangenomics and multi-omics integration and AI-driven predictive modeling,

## 3 Literature review

In the literature on rice, maize, wheat, barley, potato, cassava and yam, some information exist on genetic enhancement of selected root, tuber and cereal crops through pangenomics and multi-omics integration and AI-driven predictive modeling. Thus, this section synthesizes existing studies on the genetic enhancement of selected root, tuber and cereal crops through pangenomics and multi-omics integration and AI-driven predictive modeling.

### 3.1 Concepts of artificial intelligence, pangenomics, multi-omics, precision agriculture, and personalized breeding

Artificial intelligence (AI) encompasses two main approaches, the “imitative” perspective, which seeks to replicate human cognitive functions inspired by neural networks but struggles with the ambiguity of natural intelligence, and the “computational” approach, defining intelligence as the computational capacity to achieve goals via heuristic learning programs, a principle rooted in symbolic logic and computer science (Rozin, 2023). AI systems integrate machine learning, neural networks, and deep learning to learn from data using supervised, unsupervised, and reinforcement learning techniques, enabling autonomous adaptation and complex task performance, from virtual assistants to autonomous vehicles (Kalota, 2024). Historically, the evolution of AI has included initial optimism in the mid-20th century, setbacks dubbed “AI winters” due to unmet expectations, and resurgence fueled by big data, enhanced computational power, and refined algorithms, now reaching an era dominated by generative AI models like a generic reference to contemporary large language and vision models; focus on plant relevant AI classes (kernel methods, GBLUP/rrBLUP vs. DL; graph neural networks for pangenomes) that advance language understanding and image generation (Yang et al., 2019; Belk et al., 2023; Zhang et al., 2024). AI’s multidimensional impact spans automation, improved decision-making, personalized services, and innovation across diverse domains, making it a transformative force reshaping human-technology interaction (Fu et al., 2025; Kalota, 2024; Rozin, 2023).

Pangenomics is a genomic framework that captures the full genetic repertoire of a species by integrating all genes present across its strains or individuals, moving beyond the limitations of a single reference genome. First introduced by Tettelin et al. (2005) through the analysis of eight Streptococcus agalactiae isolates. The pangenome is typically divided into three components, the core genome (genes present in all strains, often essential for basic cellular functions), the accessory (or shell) genome (genes shared by a subset of strains, linked to environmental adaptation), and the cloud (or strain-specific) genome (genes unique to individual strains) (Tettelin et al., 2005; Golicz et al., 2020). This concept highlights that a species’ total genetic content often exceeds any individual genome and may be “open” continuously expanding with new sequenced strains, as seen in *Escherichia coli* or “closed,” where gene content saturates after few genomes, as in *Bacillus anthracis* (Aggarwal et al., 2022). Initially applied to prokaryotes, pangenomics has expanded to eukaryotes, including crops and humans, revealing roles for mobile genetic elements and structural variations in genome dynamics (Golicz et al., 2020). Modern pangenomes are constructed using gene-based presence–absence models or graph-based sequence alignments, improving variant detection, phenotype-genotype mapping, and evolutionary inference (Matthews et al., 2024). Thus, pangenomics provides a more comprehensive, unbiased view of genetic diversity, crucial for understanding adaptation, speciation, and functional genomics.

Multi-omics is an integrative systems biology strategy that combines data from multiple molecular layers such as genomics, transcriptomics, proteomics, epigenomics, metabolomics etc. to provide a comprehensive view of biological processes and their regulation (Zhang et al., 2022; Hayes et al., 2024). Unlike single-omics approaches, which often reveal only correlative associations, multi-omics enables the tracing of biological information flow from genetic variation through regulatory and functional intermediates to phenotypic outcomes (Hasin et al., 2017). For instance, integrating genomics with transcriptomics helps prioritize causal genes at disease-associated loci, while adding proteomics and metabolomics captures post-transcriptional dynamics and metabolic states closer to the phenotype (Zhang et al., 2022; Krassowski et al., 2020; Dogramaci et al., 2026). This layered integration is especially powerful in elucidating complex traits in plants and humans, revealing how genetic and environmental factors interact to shape stress responses, disease mechanisms, or agronomic performance (Roychowdhury et al., 2023). The history of multi-omics began with genomics in the 1920s by Hans Winkler in 1920, expanding in the 1980s–1990s alongside technological advances (Hasin et al., 2017). As bioinformatics tools improved, terms like proteomics and metabolomics became mainstream, organizing vast biological data. Widespread integration started in the 2000s, enabling systems-level analysis, data-driven breakthroughs, and revolutionizing molecular breeding, disease research, and precision agriculture (Zhang et al., 2022; Hayes et al., 2024). Advances in high-throughput technologies and computational methods including machine learning and network modeling have made it feasible to manage data heterogeneity, reduce dimensionality, and infer causality across omics layers (Krassowski et al., 2020). However, successful multi-omics studies require careful experimental design, adequate sample sizes, harmonized data preprocessing, and adherence to FAIR (Findable, Accessible, Interoperable, Reusable) data principles to ensure reproducibility and biological relevance (Hayes et al., 2024; Krassowski et al., 2020). Ultimately, multi-omics bridges the gap between genotype and phenotype, offering a robust framework for precision medicine, crop improvement, and functional genomics.

**Figure 1:**
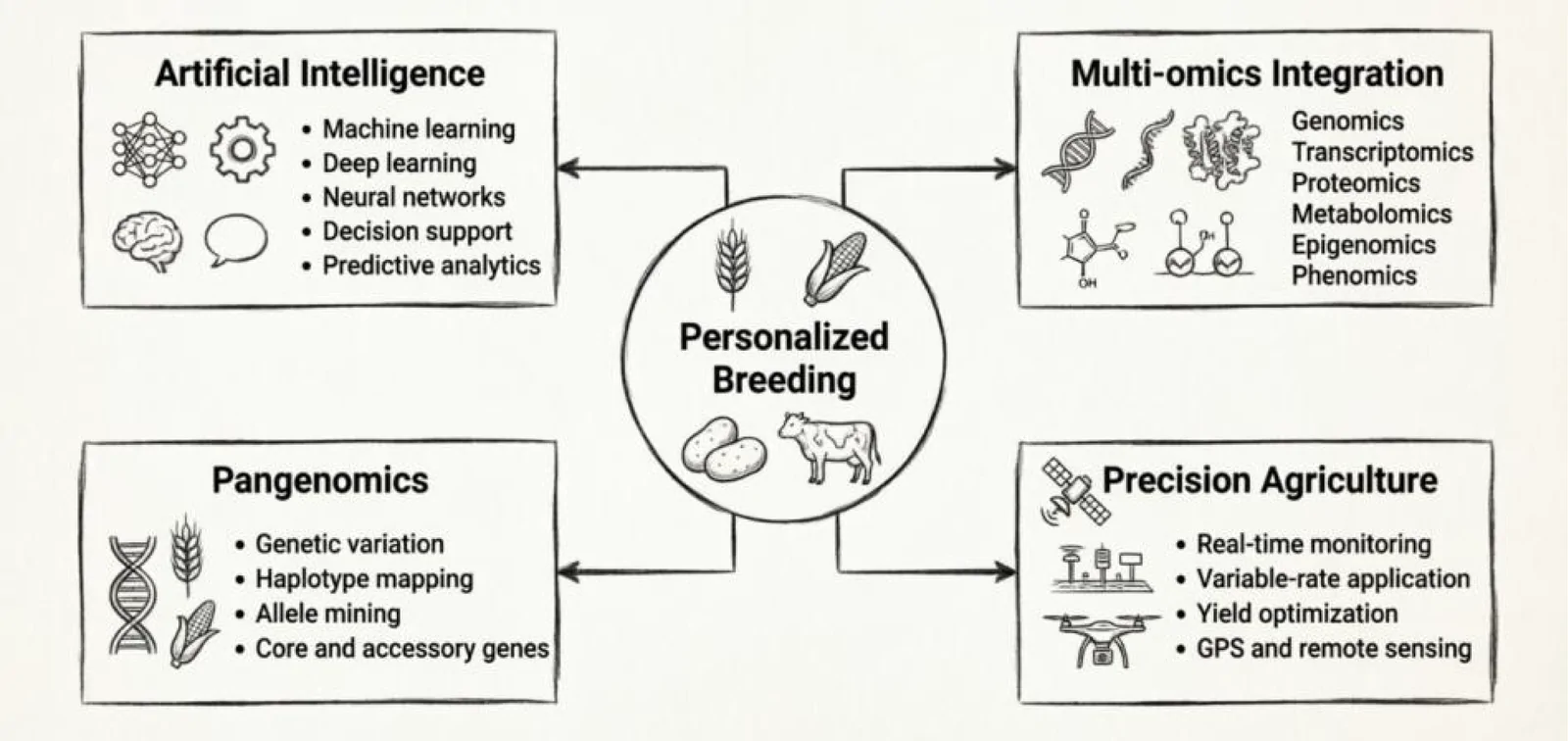
Integrated framework of artificial intelligence, pangenomics, multi-omics and precision agriculture.

Precision agriculture (PA) is a data-driven management approach that addresses spatial and temporal variability in crop production to enhance efficiency, productivity, and environmental sustainability. By integrating technologies such as Global Navigation Satellite Systems (GNSS), unmanned aerial vehicles (UAVs), remote and proximal sensors, variable rate technology (VRT), and farm management software, PA enables site-specific interventions (Shafi et al., 2019; Sishodia et al., 2020). This strategy ensures inputs like water, fertilizers, and pesticides are applied precisely where and when needed, reducing waste and minimizing ecological footprints (Getahun et al., 2024). UAVs support real-time monitoring of crop health, biomass estimation, weed mapping, and irrigation planning through multispectral imaging and vegetation indices such as NDVI (Tsouros et al., 2019). Machine learning further strengthens PA by enabling early disease detection, yield prediction, and optimized resource allocation (Sharma et al., 2021). Originally framed in the 1990s as an information-based system for whole-farm efficiency (Robert, 1999), PA now incorporates advances from multi-omics research to link biological insights such as genetic determinants of crop performance with field-scale decision-making (Fan et al., 2025). This integration of digital tools and biological knowledge positions PA as a key enabler of sustainable intensification amid climate change and rising global food demand (**Figure 1**).

Personalized breeding (PB) represents a paradigm shift in modern crop and livestock improvement, leveraging genomic selection, multi-omics integration, and precision gene-editing tools to develop varieties or breeds tailored to specific environments, production systems, and consumer preferences. By combining high-throughput phenotyping, population-specific genomic data, and advanced bioinformatics, this approach enables breeders to predict and select for complex traits such as yield stability, nutritional quality, and stress resilience—with unprecedented accuracy. Unlike conventional methods that apply generalized selection criteria, PB designs targeted strategies based on the genetic architecture and ecological context of distinct populations, accelerating genetic gain and enhancing product diversification. This strategy not only meets evolving market demands for functional foods and sustainability but also empowers farmers with high-value, locally adapted genetic material, thereby strengthening agricultural resilience and economic viability (Yang et al., 2021; Zhang et al., 2024; Zhou et al., 2017). **Table 1** summarizes the key innovations and integration points for AI, pangenomics, multi-omics, precision agriculture, and personalized breeding.

**Table 1:** Comparative overview of key innovations and integration points for AI, pangenomics, multi-omics, precision agriculture, and personalized breeding.

Concept	Key concepts	Representative tools	Primary applications	Categories/types	Reference
Artificial intelligence (AI)	Machine learning, deep learning, prediction algorithms, decision support, computer vision, NLP	AI frameworks (TensorFlow, PyTorch), machine learning models, deep learning models, smart decision support systems	Yield prediction, disease detection, genomic selection, autonomous machinery, smart decision support systems	Narrow AI, Generative AI, Supervised/Unsupervised/Reinforcement Learning, Symbolic vs. Imitative AI, CNNs, RNNs	Rozin, 2023
Pangenomics	Core/shared genes, accessory/variable genes, gene presence/absence variation, pan-genome analysis	Whole-genome sequencing (WGS), graph pangenome builders (PGGB, PanTools), comparative genomics pipelines, PAV analysis	Gene discovery, breeding for stress resilience, trait/vaccine target identification, understanding species evolution	Open vs. Closed Pangenomes, Core/Shell/Cloud Genomes, Gene-based vs. Graph-based Pangenomes	Tettelin et al., 2005; Golicz et al., 2020
Multi-omics	Genomics, transcriptomics, proteomics, metabolomics, ion omics, phenomics, top-down/bottom-up, vertical/horizontal, systems-level analysis	NGS, RNA-Seq, Mass spectrometry (LC/GC-MS), NMR, Bioinformatics platforms (COVAIN, PAINTOMICS, KaPPA-View)	Marker discovery, stress response mechanisms, nutrient-use efficiency, precision breeding targets	Top-down, Bottom-up, Vertical/Horizontal, Single-cell, Spatial Multi-Omics	Zhang et al., 2022
Precision agriculture (PA)	Site-specific crop management, variable-rate technology, IoT smart farming, remote/proximal sensing, GPS/GNSS, UAVs/drones, soil/crop sensors, GIS software	GPS/GNSS and Auto-guidance, UAVs/drones with multispectral cameras, soil and crop sensors, GIS and farm management software	Precision fertilization/irrigation, weed/pest mapping, yield monitoring, resource-use optimization	Site-Specific Crop Management (SSCM), Variable-Rate Technology (VRT), Remote/Proximal Sensing, IoT-based Smart Farming	Shafi et al., 2019; Sishodia et al., 2020
Personalized breeding (PB)	Population-personalized breeding, breeding by design, trait-specific product development, on-farm/consumer driven personalization, genomic selection, CRISPR-Cas9, high-throughput phenotyping, predictive AI models	Genomic selection (GS), CRISPR-Cas9 gene editing, high-throughput phenotyping, custom genotyping arrays, predictive AI models, multi-omics integration platforms	Locally adapted high-yielding varieties, nutrient-dense crops, climate-resilient cultivars, functional food development	Population-personalized breeding, breeding by design, trait-specific product development, on-farm/consumer-driven personalization	Yang et al., 2021; Zhang et al., 2024; Zhou et al., 2017

### 3.2 Applications of artificial intelligence and metadata in root, tuber and cereal crop breeding

The application of artificial intelligence (AI) and metadata analytics is revolutionizing breeding programs for root, tuber, and cereal crops by significantly enhancing the accuracy, efficiency, and speed of trait prediction and selection processes. The application of AI in agricultural research is rapidly shifting breeding paradigms from traditional, labor-intensive methods to data-driven, high-throughput systems. AI techniques including machine learning (ML), deep learning (DL), and data mining are now effectively processing voluminous and complex phenotypic, genotypic, environmental, and management metadata to unravel the multifaceted architecture of agronomic traits (Zhu et al., 2025; Mushtaq et al., 2024).). The studies reviewed here collectively demonstrate that AI, spanning ML and DL, offers transformative potential across the entire crop improvement pipeline, from phenotyping and disease detection to yield prediction and resource optimization (Polder et al., 2025), though significant challenges in deployment and generalization remain (**Tables 2** and **3**). Such advances allow breeders to more precisely target complex traits such as yield, disease resistance, tuber size, and stress tolerance across diverse crops like potato, cassava, yam, maize, rice, and wheat (Rana et al., 2025).

**Table 2:** Multimodal AI tools for crop phenotyping, yield forecasting, and quality assessment.

Crop (species)	Trait/focus	Site/seasons/dataset	Model/data type	Train/test setup	Main metrics and baselines	Reference
Barley	Agro-morphological diversity & Harvest Index (HI) prediction	OTGB, Ankara, Türkiye; 445 six-row accessions from 27 countries; 22 traits (11 quantitative + 11 qualitative); augmented block design, 2019/20 season	PCA, Ward’s clustering; ML, XGBoost, MARS, GP for HI prediction from 9 agronomic traits; Grid Search CV (5-fold) for hyperparameter tuning	70/30 train/test split; 5-fold CV during tuning; single-site phenotyping; diverse genetic origins (27 countries) provide internal variation	Diversity, 7 clusters; Clusters 5-7, high-yield traits (early maturity, high NGS, TGW); HI prediction, XGBoost best, RMSE = 0.137%, MAPE = 0.222%, R²=0.998 (test); top predictors, SGW, NGS, DFL	Akdogan et al. (2025)
Wheat	Early yield prediction (kg/da) using NDVI, ET, P, LST from global datasets	Ankara province, Türkiye; 25 districts, 2005-2023; TurkStat yield data + GIMMS GLAM NDVI, FLDAS ET, AgERA5 P, MODIS LST	AI algorithms, SVR, RF, ANN, LSTM; 45 scenarios (15 variable combos × 3 temporal approaches); grid/random search hyperparameter tuning; Shapley values for variable importance	70/15/15 train/val/test split; district-level spatial resolution; multi-year temporal coverage; no cross-province validation	LSTM best, R² = 0.958 (Model-19, N10_3 + LST10_3), predictions ≈3 months pre-harvest; LST most influential variable; ANN R² = 0.863, RF R² = 0.835, SVR R² = 0.758	Akcapınar and Apaydin (2025)
	Plot-level growth stage ID and flowering date prediction	China, UK, USA wheat trials; 2018–2021 drone + 2021–2022 smartphone; 70,410 canopy images with climate data	GSP-AI multimodal DL, image branch + climate time-series branch	70/20/10 train/test/val (images); cross-site within 3 countries; smartphone season as held-out test; baselines, standard vision-only CNNs	Growth stages, 91.2% (drone), 93.4% (phone); flowering RMSE, 5.6 and 4.7 days; R² up to ≈0.8; GSP-AI > all CNN baselines (≈+5–6% accuracy, lower RMSE); no CIs reported	Shen et al., 2024
Wheat	District-level yield forecasting integrating satellite imagery, meteorological data, and soil characteristics	Southern Pakistan; district-level detrended yield data 2017–2022; satellite (MODIS, Sentinel-2), meteorological (temp, precip, radiation), soil (texture, organic matter, pH) via Google Earth Engine	DeepAgroNet, three-branch DL framework—CNN for spatial features (satellite), RNN for temporal patterns (meteorology), ANN for static inputs (soil); data integration via GEE; detrending to remove long-term trends	Training, 2017–2021; Testing, 2022; validation via benchmarking against Crop Report Services; district-level modeling across southern Pakistan; no explicit cross-region validation	CNN best, R² = 0.77, Forecast Accuracy = 98% (1 month pre-harvest); RNN, R² = 0.72; ANN, R² = 0.66; All models, yield error <10%; scalable framework adaptable to other regions/crops	Ashfaq et al. (2025)
Rice	Automatic detection of 3 seedling growth stages (BBCH11, 1-leaf, BBCH12, 2-leaf, BBCH13, 3-leaf) using UAV RGB canopy images	Research Centre Shapu, Zhaoqing, Guangdong, China; 1,427 original UAV images (3-m flight height, GSD = 0.08 cm/px) across 3 cultivars, 5 densities; expanded to 49,840–90,759 images/class after cropping at 100×100 px	Traditional ML, HOG feature extraction + SVM (medium Gaussian kernel); Deep learning, EfficientNet (B0-B7) with transfer learning; compared with VGG16, ResNet50, DenseNet121; Adam optimizer, grid search for HOG hyperparameters	60/20/20 train/val/test split; internal diversity via 3 cultivars, 5 densities, multiple sowing dates; no cross-site validation	EfficientNetB4 best, Accuracy = 99.47%, Precision = 99.53%, Recall = 99.39%, F1 = 99.46%; HOG-SVM, 84.9% accuracy; EfficientNetB4 inference, 19 s for full test set; most confusions between adjacent stages (BBCH12↔BBCH13)	Tan et al. (2022); Ilo et al. (2025)
Rice	Classification of drought tolerance levels (tolerant, moderate, susceptible) using integrated physiological (RWC, H_2_O_2_, MDA, chlorophyll) and root anatomical traits (endodermis, cortex, vascular cylinder)	Greenhouse at Nakhon Phanom University, Thailand; 20 landraces + 2 reference checks; 132 observations (3 replicates × 10 plants × 22 genotypes × 2 treatments, well-watered vs. 20% PEG 6000 drought)	Random Forest (Gini/permutation importance); MLP (3 hidden layers, BatchNorm, LeakyReLU, Dropout); SHAP-optimized stacking ensemble (RF, XGBoost, SVM, MLP base learners + logistic meta-learner); SMOTE for class balancing; StandardScaler normalization	Stratified 80/20 train-test split; 5-fold cross-validation for stability; internal diversity via 20 diverse landraces; no cross-site validation	Stacking ensemble (single-split), Accuracy = 81.81%, Macro F1 = 81.2%, ROC AUC = 0.91 (tolerant/susceptible); RF (5-fold CV), Accuracy = 0.546 ± 0.067, ROC AUC = 0.730 ± 0.033; Top predictors, H_2_O_2_, RWC, endodermis inner circumference, vascular cylinder thickness	Gunnula et al. (2025)
Maize	Genomic selection for Days to Tasseling (DTT) & Plant Height (PH); parent selection guidance	NCII6210 (CUBIC), 6,210 F1 hybrids across 5 Chinese provinces; 4.5M imputed SNPs; 26 smartphone videos for phenotyping	5K functional gene-chip genotyping; smartphone 360° video + 3D Gaussian splatting; 5 ML + 4 DL models (SVM, XGBoost, RF, DeepGS, etc.); CNN+MLP environmental module	8:1:1 train/val/test; cross-province validation; phenotype validation vs manual measurements	Phenotype extraction, PH MAE = 1.53 cm, R>0.95; GS, R>0.75 (DTT), R>0.80 (PH) at N≥2,000; Parent selection, top hybrid ranked 1st-3rd in 4/5 provinces	Wu et al. (2025)
	Hybrid-specific yield prediction integrating BLUP breeding values + meteorological data	Huang-Huai-Hai Plain, China; 64 locations, 57 hybrids, 2016-2019; 2,096 observations; 19 meteorological variables	BLUP for breeding values; 4 ML models (RF, XGBoost, SVR, GPR); grid search + 10-fold CV; %IncMSE feature importance; partial dependence profiles	80/20 train/test split; multi-location internal validation; future projections (2025-2054)	Best, RF achieved R²=0.64, RMSE = 1,010.6 kg/ha, MAPE = 8.25%; Key predictors, breeding value > radiation > water variables; Thresholds, TMAX>31 °C, PRECTOT>5 mm/day reduce yield	Wang et al. (2025a)
Potato	Tuber size, shape, skin/flesh color, hollow-heart defect	Aberdeen, Idaho (USA); 2019; 189 F1 progeny + parents; 1,890 whole-tuber image sets; 2,431 sliced-tuber images (augmented)	Low-cost RGB imaging + OpenCV/PlantCV for size/shape/colour; CNN for hollow-heart classification	Single site; 10 tubers/clone; hollow-heart CNN, ~80/20 train/test with augmentation; multiple image resolutions tested	Size and shape, r² > 0.93 vs manual; heritability moderate–high. Hollow-heart CNN (512×512), ≈99% test accuracy, very low loss, fast inference (~3 ms/image); no CIs reported	Feldman et al., 2024
	Field-scale tuber yield under N rates	Oregon, USA; 2020–2021; 264 plots; weekly UAS multispectral over 5 stages	CAR Conv1D-BiGRU-BiLSTM-Net on time-series VIs + N-rate; linear unmixing + Otsu for vegetation features	80/20 train/test (plots); 2 seasons combined; single-site only; baselines, simpler deep models (Conv1D, BiGRU, BiLSTM, etc.)	Best setup (5 indices + N, stages T1–T4), R² ≈ 0.78, RMSE ≈ 8.35×10³ kg/ha (≈16.4%); outperforms all tested DL baselines; CIs not reported	Yadav et al., 2025
Cassava	Estimation and forecasting of blue (WFb) and green (WFg) water footprints using meteorological variables and crop parameters	Nanning city, Guangxi, China; 14 weather stations; monthly data 1994–2019 (Tmax, Tmin, P, WS, SH, H, Kc, yield); CROPWAT 8.0 for WF calculation; 80/20 temporal split (train, 1994–2014, test, 2015–2019)	AI models, ANN (2 hidden layers, ReLU, SGD), SVM (RBF/PK/LK kernels), RF (ensemble trees); SARIMA for meteorological forecasting; PCA for input selection; grid search for hyperparameters; metrics, RMSE, NRMSE, R², ACC, NSE	Held-out temporal test set (2015–2019); 5-fold CV not reported; cross-site generalization not evaluated; SARIMA residuals tested for white noise; potential transferability to similar subtropical monsoon regions noted	WFb, ANN7 (Tmax,Tmin,P,Kc,H,SH; layers 8,6), R²=0.9499, RMSE = 4.69 m³/ton, ACC = 97.16%; WFg, ANN14 (Tmax,Tmin,P,WS,SH; layers 7,5), R²=0.9258, RMSE = 4.25 m³/ton, ACC = 96.21%; SARIMA forecast R²=0.82–0.95; AI-SARIMA coupled prediction deviations <6%	Tao et al. (2023)
Sweet Potato	Yield prediction (#1 Boxes, Tot Mkt) using UAV multispectral imagery, vegetation indices (VIs), and physiological parameters under varying N rates and cover crops	Pontotoc Ridge-Flatwoods Exp. Station, Mississippi, USA; 60 plots × 9 UAV flights (Jun–Sep 2022); 540 observations, 21 features (spectral bands, VIs, chlorophyll, biomass, vine length, leaf N)	ML models, Lasso, SVR, RF, XGBoost; soft-voting ensemble; feature selection via Kendall Tau, VIF (<15), RF importance; key VIs, SCCCI, CIRE, NGRDI; grid search + 10-fold CV for hyperparameters	80/20 random train/test split; treatment-level subgroup analysis for internal robustness; no cross-site validation; cross-validation during tuning (5-fold for RF, 10-fold for ML)	Physiological features, KNN best (R²≈0.85, RMSE≈80 boxes); VI-only, XGBoost/RF R²=0.61–0.65, RMSE = 110–114 boxes; Cover crop > N treatment effect; SCCCI, CIRE top VIs; ensemble R²=0.65–0.70 across treatment subgroups	Singh et al. (2025)
	Prediction of flesh-color intensity and mealiness by hand for boiled roots using RGB image analysis	Uganda; CIP breeding team; 217 phenotypes, 1,487 images from 950 samples (boiled after peel/cross-section); 227 ground truth samples from trained sensory panel (10–13 experts)	Flesh-color, regression (LR, KNN, DT, SVM, RF) + classification (DT, RF) using RGB/HSV/LAB pixel features; Mealiness, RF, XGBoost, custom NN using CNN-extracted features (MobileNetV3, EfficientNet); background extraction via YOLOv5 + K-means	80/20 random train-test split; class imbalance (70% samples in 0–5 flesh-color range); no cross-site validation; noted need for region-specific retraining (e.g., Mozambique)	Flesh-color, Linear Regression R² = 0.92, MSE = 0.58; Mealiness, Random Forest R² = 0.85, MAE = 0.90; Web tool, ~2.2 s/image (flesh-color), ~5.6 s/image (mealiness); ~95% time reduction vs. sensory panel (30 min/sample)	Nakatumba-Nabende et al. (2023)
Yam	Classification of yam quality (Good, Diseased Damaged, Insect Damaged) using acoustic properties from two sound-generation techniques	Nigeria; National Root Crops Research Institute, Umudike; 600 tubers (300 white, 300 yellow); 100 Good, 100 Diseased, 100 Insect per species; acoustic properties, amplitude, frequency, intensity, period, velocity, wavelength	Two devices, (1) Software Sound Generation (200 Hz fixed frequency, speaker + microphone); (2) Surface Impact Sound Generation (yam impact on surface); Multiple Discriminant Analysis (MDA) with leave-one-out cross-validation; Wilks’ Lambda for feature selection	Leave-one-out cross-validation; no cross-site validation; noted need for basic acoustic/computer knowledge by users; potential barriers in resource-limited settings	White yam, Software technique accuracy = 79.0% (CV), Impact = 68.7%; Yellow yam, Software = 82.3%, Impact = 68.7%; Software technique better for Good yams (68–87%), Impact better for damaged classes (up to 100%); Software required fewer acoustic variables (1–4 vs. 4–5)	Audu et al. (2023)
Carrot	Yield prediction (total fresh mass, root mass) and quality attributes (°Brix, firmness) using satellite-derived vegetation indices	São Gotardo, Minas Gerais, Brazil; two commercial fields; 200 georeferenced sampling points (30×30 m grid) at 82 and 116 DAS; PlanetScope CubeSat imagery (3 m resolution)	ML regression, ANN, RF, MLR; predictor variables, NDVI, SAVI, EVI, RDVI; PCA for feature selection; normalization; stratified 70/30 train-test split by sampling period; cross-validation during training	70/30 train-test split stratified by 82 vs. 116 DAS; cross-site comparison (Site 1 vs. Site 2); hailstorm at Site 1 introduced environmental variability; no external validation	ANN best, R² = 0.68, RMSE = 23.80 boxes ha^-^¹ (1 box = 29 kg); RF, R² = 0.67, RMSE = 23.93; MLR, R² = 0.61, RMSE = 24.21; SAVI & NDVI most predictive (r = 0.67–0.78 with root mass); Quality attributes (°Brix, firmness) not predictable (R² < 0.5)	de Lima Silva et al. (2024)

AI, Artificial Intelligence; ANN, Artificial Neural Network; AutoGP, Automatic Genomic Prediction; BBCH, Biologische Bundesanstalt, Bundessortenamt und CHemische Industrie growth stage scale; BiGRU, Bidirectional Gated Recurrent Unit; BiLSTM, Bidirectional Long Short-Term Memory; BiSeNetV2, Bilateral Segmentation Network Version 2; BLUP, Best Linear Unbiased Prediction; CAR, Context-Aware Attention + Residual Connection; CC, Crop Cover; CHLGR, Chlorophyll Green Ratio; CIRE, Chlorophyll Index Red Edge; CNN, Convolutional Neural Network; Conv1D, One-Dimensional Convolutional Neural Network; CV, Cross-Validation; CVI, Chlorophyll Vegetation Index; DFEB, Dynamic Feature Extraction Block; DFL, Days to 50% Flowering; DL, Deep Learning; DLGWAS, Deep Learning Genomic-Wide Association Study; DRFConv, Dynamic Receptive-Field Convolution; DT, Decision Tree; DTT, Days to Tasseling; EAMA, Efficient Asymmetric Multi-scale Attention; ET, Evapotranspiration; FLDAS, Famine Early Warning Systems Network Land Data Assimilation System; FLOPs, Floating Point Operations; GDD, Growing Degree Days; GBDT, Gradient-Boosted Decision Tree; GEE, Google Earth Engine; GIMMS, Global Inventory Modeling and Mapping Studies; GLAM, Global Agricultural Monitoring; GP, Gaussian Processes; GPR, Gaussian Process Regression; GS, Growth Stage; GSD, Ground Sampling Distance; H², Broad-sense heritability; H_2_O_2_, Hydrogen Peroxide; HI, Harvest Index; HOG, Histograms of Oriented Gradients; IoU, Intersection over Union; Kc, Crop Coefficient; KNN, K-Nearest Neighbors; LR, Linear Regression; LST, Land Surface Temperature; LSTM, Long Short-Term Memory; LUM, Linear Unmixing; MAD, Mean Absolute Deviation; MAE, Mean Absolute Error; MAPE, Mean Absolute Percentage Error; MARI, Modified Anthocyanin Reflectance Index; MARS, Multivariate Adaptive Regression Spline; MDA, Malondialdehyde or Multiple Discriminant Analysis; ML, Machine Learning; MLP, Multilayer Perceptron; MLR, Multiple Linear Regression; MODIS, Moderate Resolution Imaging Spectroradiometer; MSE, Mean Squared Error; N, Nitrogen; NDVI, Normalized Difference Vegetation Index; NGRDI, Normalized Green Red Difference Index; NGS, Number of Grains per Spike; NIR, Near-Infrared; NN, Neural Network; NRMSE, Normalized Root Mean Square Error; NSE, Nash-Sutcliffe Efficiency; OTGB, Osman Tosun Gene Bank; P, Precipitation; PCA, Principal Component Analysis; PDP, Partial Dependence Profile; PEG, Polyethylene Glycol; PH, Plant Height; PLSR, Partial Least Squares Regression; R²/r², Coefficient of Determination; ReLU, Rectified Linear Unit; RF, Random Forest; RGB, Red–Green–Blue; RMSE, Root Mean Square Error; RNN, Recurrent Neural Network; RWC, Relative Water Content; SARIMA, Seasonal Autoregressive Integrated Moving Average; SAVI, Soil Adjusted Vegetation Index; SCCCI, Simplified Canopy Chlorophyll Content Index; SGW, Spike Grain Weight; SHAP, Shapley Additive Explanations; SMOTE, Synthetic Minority Over-sampling Technique; SNP, Single-Nucleotide Polymorphism; SPAM, Spatial Production Allocation Model; SR, Simple Ratio; SVM, Support Vector Machine; SVR, Support Vector Regression; TCARI, Transformed Chlorophyll Absorption Reflectance Index; TGW, Thousand-Grain Weight; UAS, Uncrewed Aerial System; UAV, Unmanned Aerial Vehicle; VF, Vegetation Fraction; VI, Vegetation Index; VIF, Variance Inflation Factor; VIP, Variable Importance in Projection; WFb/WFg, Blue/Green Water Footprint; WGSP, Wheat Growth Stage Prediction dataset; WI, Willmott Index; XGBoost, Extreme Gradient Boosting; YOLO, You Only Look Once.

**Table 3:** AI applications for crop disease detection, segmentation, and resistance prediction.

Crop	Trait/focus	Site/dataset	Methods	Train/test & generalization	Key results	Reference
Barley	In-field detection of NFNB, SFNB, Scald	DPIRD, WA; 926 patches (448×448 px) from 312 RGB images; 4 classes	Transfer learning, ResNet50/101, DenseNet121, InceptionV3, Xception, MobileNet; Adam optimizer; augmentation (flips, rotations)	10-fold CV; 80/20 train/val within folds; augmentation on training only	MobileNet best, Binary acc = 98.63% avg; 4-class acc = 93.50%, F-score = 93.49%, AUC = 97.55%; 3M params, 4.36 ms inference	Rezaei et al. (2024)
	Net blotch (Pyrenophora teres) necrosis area quantification	Controlled environment, Portugal; cultivar ‘Siberia’; 1,800 RGB smartphone images (64 MP) at 0, 2, 4, 7, 10 dpi; 3 biological replicates	Two-stage DL, Cascade R-CNN (ResNet50) for leaf detection + U-Net for necrosis segmentation; augmentation; qPCR validation (3 fungal genes)	80/20 train/test split; 3 biological repetitions; temporal validation across 5 infection timepoints	Cascade R-CNN, 98.3% accuracy, F1 = 95%; U-Net, 99% accuracy, Dice = 0.99, Jaccard = 0.98; Latency, 0.15–0.3 s/image; DL vs. manual/qPCR, R² = 0.96	Bouhouch et al. (2024)
	Few-shot learning for disease detection with limited labels	Same DPIRD site; 2,165 patches; 5 classes (incl. early/severe NFNB)	FSL pipeline, Swin-T/B, ViT-B, ResNet50 backbones + prototypical networks; SGD, warm-up + cosine LR; GradCAM	5-way 1/5-shot episodic training; 65/15/20 split; 600 episodes; cross-dataset validation on Cassava	Swin-B best, Barley, 86.78% (1-shot), 91.88% (5-shot); Cassava, 97.93% (1-shot), 95.24% (5-shot); meta-training essential	Rezaei et al. (2025)
	Powdery mildew (AUDPC) resistance prediction from phenotypic traits + molecular markers	Gonbad Kavous Univ. Farm, Iran; F8–F9 biparental population (≈130 lines from ‘Badia’ × ‘Kavir’); 3 sowing dates × 2 seasons; 15 traits + 719 markers	Feature selection, RReliefF, MRMR, F-test. ML, Decision Tree, Random Forest, Neural Network (NET), Gaussian Process Regression, with Bayesian hyperparameter tuning	70/30 train/test split; 5-fold CV on train; performance by MAE, RMSE, R²; Friedman test for pairwise model comparison	NET + MRMR/RReliefF gave lowest MAE and RMSE and highest R² on AUDPC, slightly improved by combining phenotypic and genotypic data; some overfitting but overall strong predictive ability	Vahidpour et al., 2025
Wheat	Detection of 14 wheat diseases using symptom-based text descriptions	Pakistan; multi-source dataset (farms, literature, University of Agriculture Faisalabad); 43 curated instances, 14 diseases, 4 symptom features (plant part, color, appearance)	ML classifiers, Decision Tree, Random Forest, SVM, AdaBoost; Feature extraction, Count Vectorization (CV), TF-IDF; Info gain for feature selection; Grid search for hyperparameter tuning	80/20 train/test split; expert validation; no cross-validation reported; data from diverse Pakistani sources for internal variation	SVM+CV best, Accuracy = 99%, Precision = 95%, Recall = 98%, F1 = 96%; CV generally outperformed TF-IDF; DT/RF stable at 85–86% accuracy; AdaBoost 91% with CV; mobile app with offline functionality deployed	Niaz et al. (2025)
Maize	Real-time detection & classification of 3 diseases (GLS, NLB, MSV) + healthy; severity assessment & treatment advice	Zambia Maize Leaf Dataset (ZMLD), 19,990 field-captured images across 4 provinces; 4 classes; YOLO-format bounding boxes	ZamYOLO-Maize, YOLOv8n backbone (C2f+SPPF), PANet+FPN neck, decoupled head; CIoU+BCE loss; hierarchical CNN for classification; rule-based treatment engine	70/20/10 stratified split; data augmentation (rotation, brightness, flipping); multi-province collection for internal variability; no cross-country validation	YOLOv8n, Precision=0.991, Recall=0.997, F1 = 0.995, mAP@50 = 0.995, inference=4.65 ms/image; YOLOv10s, F1 = 0.999 but slower (8.24 ms); robust to field variability	Kalunga et al. (2026), Front. Artif. Intell. 9:1764283
	Lightweight, interpretable CNN classification of 4 disease categories (Healthy, GLS, Common Rust, NLB)	Kaggle ‘Corn or Maize Leaf Disease Dataset’, 4,062 field-condition JPG images; 4 classes; slight imbalance (GLS, 513)	Custom 3-stage CNN (64/128/256 filters), BatchNorm, MaxPool, Dropout; Global Avg Pooling; Adamax optimizer; XAI, LIME & SHAP for interpretability	80/10/10 random split; augmentation (rotation, flip, zoom, brightness, shear); robustness tested via 5 simulated stress scenarios	Accuracy=94.97%, Micro-AUC=0.99, F1 = 0.94; Params=1.22M, Inference=57.85 ms; SHAP IoU=0.82; outperformed ResNet50, MobileNetV2, EfficientNetB0	Alpsalaz et al. (2025)
	Classification of 4 leaf diseases (NLB, CR, GLS, Healthy) using hybrid CNN-ViT architecture	Kaggle (4,188 images, lab-controlled) + Mendeley (3,852 images, field-collected Ghana); combined, 8,040 images; external validation, CD&S dataset (1,597 field images, Purdue)	Hybrid CNN-ViT, CNN branch (6 Conv2D blocks) for local features; ViT branch (6 transformer layers, 16×16 patches, 8 heads) for global context; feature concatenation + dense classifier; RAdam optimizer; Grad-CAM for interpretability	80/10/10 stratified split; 5-fold cross-validation; external validation on independent CD&S dataset; augmentation (flip, rotation) for class imbalance	Hybrid CNN-ViT, Accuracy = 99.15%, Precision = 99.13%, Recall = 99.13%, F1 = 99.12%; CD&S generalization, 95.93% accuracy; ECE, 0.0015–0.0351; Params, 87.05M, Inference, 29.7 ms	Shandilya et al. (2025)
Rice	Classification of 7 rice diseases using DenseNet121 with transfer learning	Paddy-Rice Dataset (open-source); 8,030 original images → 11,467 after augmentation; 7 classes (Bacterial Leaf Streak, Brown Spot, Tungro, Bacterial Leaf Blight, Blast, Downy Mildew, Bacterial Panicle Blight)	DenseNet121 (121 layers, 4 dense blocks) with ImageNet pre-trained weights; transfer learning (freeze/unfreeze strategy); Global Average Pooling, Dropout, Dense classification head; Adam optimizer; ReduceLROnPlateau scheduler	80/20 random split with class balancing; 5-fold cross-validation; real-time augmentation (rotation, zoom, flip, brightness) during training	DenseNet121, Accuracy = 97.9% (test), 97.8% ± 0.42% (5-fold CV); 95% CI, Accuracy [97.2%, 98.4%], F1 [96.6%, 97.6%]; Class-wise F1, 96–97%; Params, ~8M	Ismail et al. (2026)
Wild Rice	Rice blast disease level evaluation (L0-L9) and resistance identification (R0-R9) via lesion segmentation	1,045 wild rice germplasm from 5 Chinese provinces; 7,100 augmented images (1,420 original) captured at 4–7 dpi using Sony DSLR, AR glasses, high-speed scanner; annotated with LabelMe	Improved YOLOv8-Seg, GhostHierarchicalNet backbone, CAHSFPN neck, LSDECS head with DEConv; LAMP pruning (82% params reduction); FAGH federated learning for non-IID data; LCR-based disease level classification	80/10/10 train/val/test split; cross-site validation at Anhui Academy (100 cultivated rice materials); practical testing, 100 wild rice × 3 replicates; federated learning across 4 edge devices	Segmentation, mAP@0.5 = 96.3%, Params = 0.22M, FLOPs = 5.3G; Disease evaluation, 99.7% accuracy (test), 99.0% (practical); Identified 3 highly resistant materials (#002, #025, #036); Generalization, 96.0% on cultivated rice	Pan et al. (2025)
Cassava	Detection and localization of 5 leaf conditions (CBB, CBSD, CGM, CMD, Healthy) using object detection models	Uganda; farmer-collected smartphone images; 2,822 annotated images (bounding boxes via Roboflow); 5 classes; balanced distribution; augmentation (flip, rotation, noise)	YOLOv9-c/e and GELAN-c/e models; transfer learning from MS COCO; PGI for gradient preservation; GELAN for efficient layer aggregation; PyTorch, Tesla P100 GPU; grid search hyperparameters	Fixed 2,469/235/118 train/val/test split; augmentation to address limited samples; no cross-site validation; potential for mobile deployment in field conditions noted	All models, Precision = 75%, Recall = 80–83%, mAP@0.5 = 83–86%; vs. YOLOv8, +5 pp Precision, +8 pp Recall, +8–11 pp mAP@0.5; Best classes, CGM/CMD (88–93% correct); Worst, CBB (51–65%) due to feature variability	Kalezhi and Shumba (2025); Ceballos and Fenstemaker, 2026
Sweet Potato	Classification of water stress levels (5-class, SD/D/O/W/SW; simplified to 3-class, SD/O/SW) using low-altitude RGB-thermal imagery + growth indicators	Gyeongsang National University, South Korea; Jinyulmi cultivar; 2 plots (320 m² each); ~300 samples (50/treatment); 2,374 leaf temp values, 632 thermal + 452 RGB images after filtering; VWC-based stress levels (SD ≤10%, D 20 ± 2%, O 30 ± 3%, W 40 ± 3%, SW ≥50%)	ML, LR, RF, KNN, MLP, SVM with growth indicators (stem length, NDVI, CF, SPAD, CWSI); DL, CNN-ViT fusion model (~220K params); redefined CWSI using field-measurable variables (Tc, Ta, RH, soil moisture); Grad-CAM for XAI; GUI with Tkinter/OpenCV	80/20 stratified train-test split; 5-fold cross-validation (random_state=42); stage-specific analysis (June/July/August); no cross-site validation; class consolidation (5→3) to improve DL performance	KNN (ML), Accuracy = 1.0 (all stages), 5-fold CV, 0.94–0.96; CNN-ViT (DL), 5-class acc = 0.75; 3-class acc = 0.92 (CV, 0.91), SD/SW = 100%, O = 97%; Top features, CWSI, leaf temp, stem length, SPAD; ANOVA, F = 12.57, p = 0.043	Choi et al. (2025)
Taro	Early detection of Taro Leaf Blight (TLB) using object detection on taro leaf images	Nigeria (Ebonyi, Enugu) + Ghana (Ashanti); 13,887 smartphone-captured images; 3 classes, Taro_Early_Blight (5,200), Taro_Healthy (4,300), Taro_Not_Early (4,387); 70/20/10 train/val/test split	YOLOv8 fine-tuned with transfer learning; C2f+SPPF backbone, PAN-FPN neck, decoupled head; BCE + CIoU+DFL loss; Adam optimizer; GPU training (NVIDIA T4); Android app with FastAPI backend + Cloudinary storage	Fixed 9,711/2,784/1,392 split; field testing with 42 participants across Nigerian states; cross-site assessment via Nigeria/Ghana data; noted Android phone ownership limitations (40% of farmers)	Training B (13,887 images), mAP@0.5 = 0.857 overall, Early class mAP = 0.822, Precision = 0.86, Recall = 0.81, F1 = 0.83; Inference, ~50 FPS on GPU; App usability, 87% user-friendly, 78% easy to understand, 64% accurate detections, 75% believe early TLB detection possible	Nwaneto et al. (2025)
Yam	Segmentation of leaf structures and disease lesions (anthracnose, brown spot, gray spot) in complex field conditions	Jiaozuo, Henan, China; 1,097 original images (396 anthracnose, 363 brown spot, 338 gray spot) → 7,679 after augmentation; Canon EOS 70D + iPhone XR; pixel-level annotation via LabelMe	BiSeNeXt, improved BiSeNetV2 with DFEB (DRFConv+PixelShuffle), EAMA (asymmetric multi-scale attention), PointRefine decoder; CE loss, SGD optimizer; data augmentation (saturation, brightness, crop, rotation, flip)	7:2:1 train/val/test split; cross-crop validation on Apple Leaf Dataset (1,222 images) without fine-tuning; no cross-site validation within yam dataset	BiSeNeXt, Leaf IoU = 97.04%, Disease IoU = 84.75%, mIoU = 93.62%, mF1 = 96.59%; Params = 3.22M, FLOPs = 20.82G, Inference = 18.62 ms; outperformed DeepLabV3+, UNet++, Swin Transformer; Apple dataset generalization, Disease IoU = 80.66%	Lu et al. (2025)

AdaBoost, Adaptive Boosting; ANN, Artificial Neural Network; AR, Augmented Reality; AUC, Area Under ROC Curve; AUDPC, Area Under Disease Progress Curve; BCE, Binary Cross-Entropy; BiSeNetV2, Bilateral Segmentation Network Version 2; CAHSFPN, Context-Aware Hierarchical Scalable Feature Pyramid Network; CART, Classification and Regression Tree; Cascade R-CNN, Cascade Region-based Convolutional Neural Network; CBB, Cassava Bacterial Blight; CBSD, Cassava Brown Streak Disease; CF, Chlorophyll Fluorescence; CGM, Cassava Green Mite; CIoU, Complete Intersection over Union; CMD, Cassava Mosaic Disease; CNN, Convolutional Neural Network; CR, Common Rust; CV, Count Vectorization; CWSI, Crop Water Stress Index; DEConv, Detail Enhancement Convolution; DFEB, Dynamic Feature Extraction Block; DFL, Distribution Focal Loss; DiC, Difference in Count; Dice, Segmentation overlap metric; DL, Deep Learning; DPIRD, Department of Primary Industries and Regional Development; dpi, Days Post-Infection; DRFConv, Dynamic Receptive-Field Convolution; DT, Decision Tree; EAMA, Efficient Asymmetric Multi-scale Attention; ECE, Expected Calibration Error; F1, Harmonic mean of precision and recall; FAGH, Federated Averaging with Gradient Harmonization; FLOPs, Floating Point Operations; FPS, Frames Per Second; FSL, Few-Shot Learning; GELAN, Generalized Efficient Layer Aggregation Network; GLS, Gray Leaf Spot; GPR, Gaussian Process Regression; GPU, Graphics Processing Unit; Grad-CAM, Gradient-weighted Class Activation Mapping; GUI, Graphical User Interface; IoU, Intersection over Union; Jaccard, Intersection over Union metric; KDD, Knowledge Discovery in Database; KNN, K-Nearest Neighbors; LAMP, Layer-wise Adaptive Magnitude Pruning; LCR, Lesion Coverage Ratio; LIME, Local Interpretable Model-agnostic Explanations; MAE, Mean Absolute Error; mAP, mean Average Precision; ML, Machine Learning; MLR, Multiple Linear Regression; MRMR, Minimum Redundancy Maximum Relevance; MSV, Maize Streak Virus; NDVI, Normalized Difference Vegetation Index; NET, Neural Network; NFNB, Net Form Net Blotch; NLB, Northern Leaf Blight; O/SD/SW, Optimal, Severe Dry, Severe Wet; PCA, Principal Component Analysis; PCGrad, Projection Conflict Gradient; PGI, Programmable Gradient Information; qPCR, Quantitative Polymerase Chain Reaction; R², Coefficient of Determination; RAdam, Rectified Adam; RF, Random Forest; RIL, Recombinant Inbred Line; RMSE, Root Mean Square Error; RReliefF, Regression Relief Feature Selection; SAVI, Soil Adjusted Vegetation Index; SBD, Symmetric Best Dice; SGD, Stochastic Gradient Descent; SFNB, Spot Form Net Blotch; SHAP, SHapley Additive exPlanations; SPAD, Soil Plant Analysis Development; SVM, Support Vector Machine; TF-IDF, Term Frequency-Inverse Document Frequency; TLB, Taro Leaf Blight; U-Net, Encoder-decoder CNN with skip connections; VWC, Volumetric Water Content; ViT, Vision Transformer; XAI, Explainable Artificial Intelligence; YOLO, You Only Look Once.

A central theme across the literature is the use of computer vision and deep learning to automate disease diagnosis. In maize and potato breeding, AI models like Random Forest, Support Vector Machines, and Light Gradient Boosting Machine (LightGBM) have been successfully applied to predict yield and tuber quality from multispectral drone imagery and field sensor data with high accuracy (Yadav et al., 2025; Thinakaran et al., 2025). For crops like maize and potato, DL models such as convolutional neural networks (CNNs) and YOLO variants achieve high accuracy in controlled settings. Machine vision and convolutional neural networks demonstrate remarkable performance in quantifying tuber size, shape, and detecting internal defects such as hollow heart, enabling scalable, non-destructive phenotyping (Feldman et al., 2024). In potatoes, machine vision workflows can quantify tuber size, shape, and color, while also classifying internal defects like hollow-heart with high accuracy. This capacity to precisely phenotype at scale underpins rapid genetic gain and selection efficiency, specifically in root and tuber crops where traditional phenotyping is laborious and time-consuming. However, a critical issue is the significant performance drop when models are evaluated under real-field “open-set” conditions, where lighting, background, and symptom expression vary widely. This underscores the necessity of training on diverse, field-collected datasets and developing robust, generalizable architectures (Ahmed et al., 2025). Across the reviewed studies, convolutional neural networks (CNNs), particularly VGG16, ensemble YOLOv8, and custom architectures like GSP-AI, consistently achieve the highest accuracy for image-based disease detection and phenotyping when sufficient labeled data and computational resources are available.

Across the literature, deep learning (DL) consistently outperforms traditional machine learning for image-based disease detection, with CNNs and vision transformers achieving the highest accuracy. For barley, MobileNet with transfer learning proved highly effective for in-field detection of net blotch and scald, while a few-shot learning pipeline with Swin transformers addressed data scarcity by achieving strong performance with minimal labeled samples (Rezaei et al., 2024, Rezaei et al., 2025). For wheat, a text-based SVM system was deployed as an offline Android app for multiple disease detection (Niaz et al., 2025). For maize, a hybrid CNN-ViT model showed superior performance with strong generalization to field conditions (Shandilya et al., 2025), while a lightweight CNN with XAI integration provided interpretable classifications (Alpsalaz et al., 2025). ZamYOLO-Maize, using YOLOv8n, enabled real-time mobile deployment on a Zambian field dataset (Kalunga et al., 2026). For rice, DenseNet121 with transfer learning effectively classified multiple diseases (Ismail et al., 2026). For wild rice blast, an improved YOLOv8-Seg model with federated learning achieved efficient disease evaluation (Pan et al., 2025). For cassava, YOLOv9 and GELAN models outperformed earlier YOLO versions (Kalezhi and Shumba, 2025). For taro, a YOLOv8-based Android app provided field-deployable early blight detection (Nwaneto et al., 2025). For sweet potato water stress, a CNN-ViT fusion model with Grad-CAM offered interpretable classification (Choi et al., 2025). For yam, BiSeNeXt achieved efficient disease segmentation with minimal parameters (Lu et al., 2025). While models like VGG16 have shown high accuracy for specific diseases like downy mildew (Jadesha et al., 2026), ensemble methods (e.g., weighted ensembling of YOLOv8 variants) can further boost robustness (Muthulakshmi et al., 2024).

AI also excels in predicting yield and growth dynamics using environmental and genotypic data. For temporal prediction tasks such as yield forecasting and water footprint estimation, hybrid architectures combining recurrent networks (LSTM, BiGRU) with attention mechanisms prove superior (Yadav et al., 2025; Akcapınar and Apaydin, 2025; Tao et al., 2023). For rice, hybrid models like ANN-GP have outperformed stand-alone ANNs in yield estimation (Babaee et al., 2021), while models like GEP and ANN can simulate growth rates from temperature data, with ANN achieving lower RMSE (Liu et al., 2021). For cereals like wheat, DL models can classify growth stages and predict flowering time using drone imagery and climate data (Shen et al., 2024). For phenology and growth-stage monitoring, hybrid CNN+RNN models (e.g., GSP-AI with Res2Net-19 + LSTM) achieve high accuracy and low flowering RMSE by fusing drone imagery with climate time series (Shen et al., 2024). For yield forecasting, LSTM and ANN models leveraging satellite-derived vegetation indices (NDVI, SAVI) and meteorological data reach high predictive power and low error (Akcapınar and Apaydin, 2025; Ashfaq et al., 2025). These predictive tools are crucial for breeders to anticipate crop performance under varying conditions. Furthermore, AI-driven decision support systems can translate predictions into actionable advice, such as site-specific fungicide applications that have been shown to reduce disease severity and increase economic returns in maize (Jadesha et al., 2026).

The integration of genomic selection with multi-omics datasets—transcriptomics, proteomics, and metabolomics—further enriches breeding insights, facilitating causal variant discovery and elucidation of genotype-to-phenotype mechanisms amidst the polygenic complexity common in cereals and tubers (Zhu et al., 2025). AI-powered multi-omics integration notably advances the identification of superior alleles associated with resilience to abiotic and biotic stresses, supporting targeted breeding strategies for climate-adaptive cultivars (Mushtaq et al., 2024). Similarly, AI can help identify stress-tolerant traits, as demonstrated by analyses linking root architecture and hormonal profiles to drought tolerance in wheat varieties (Licaj et al., 2023). Furthermore, linking phenotypic analysis (PA) covariates (envirotyping) into genomic prediction frameworks enables AI-powered enviro genomic models that synthesize environmental and genetic data for enhanced predictive accuracy. For powdery mildew resistance prediction, neural networks combined with MRMR or RReliefF feature selection successfully integrated phenotypic traits with molecular markers (Vahidpour et al., 2025).

High-throughput phenotyping technologies, including UAV-based hyperspectral imaging and proximal sensors, coupled with AI analytics, enable dynamic monitoring of physiological and biochemical crop traits such as photosynthetic efficiency and nutrient uptake efficiency (Shen et al., 2024). Similarly, for cassava, automated image analysis frameworks like CIAT Pheno-i can extract vegetation indices from UAV multispectral imagery to predict root yield. These real-time data streams inform genotype-by-environment interaction models, capturing nonlinear and epistatic effects that traditional approaches often miss. This capability is especially critical for breeding climate-resilient wheat varieties where environmental variability profoundly influences phenotypic expression (Mushtaq et al., 2024). The application of AI is not limited to field crops. In *in vitro* systems, response surface methodology (RSM) combined with ML (e.g., Random Forest) can optimize media components (e.g., sucrose, BAP) for efficient potato tuberization, with potential for predicting optimal conditions for large-scale propagation (Thinakaran et al., 2025).

AI also supports precision agriculture applications by integrating metadata from soil sensors, climatic datasets, and remote sensing to optimize nutrient management, disease surveillance, and yield prediction, thereby enhancing decision support systems for breeders and farmers alike (Raj et al., 2025; Zhu et al., 2025). Furthermore, AI-driven predictive breeding platforms simulate numerous parental crosses to identify optimal combinations that maximize yield potential and resilience, expediting breeding cycles far beyond conventional methodologies (Farmonaut, 2025; AgrinextCon, 2025). AI-driven decision support systems can translate predictions into actionable advice, such as site-specific fungicide applications.

Despite these advances, several key limitations persist. A primary challenge is the “deployment gap”, the difficulty of moving models from controlled research environments to real-world farming conditions, where they often face data fragmentation, high computational demands, and a lack of user-friendly interfaces (Gupta and Kumar Pal, 2025; Murugan et al., 2026). Challenges remain, including high implementation costs, data interoperability complexities, ethical concerns surrounding data ownership, and limited model interpretability. While lightweight models and smartphone applications (e.g., for maize leaf disease) improve accessibility (Paul et al., 2025; Dhanaraj et al., 2025), achieving high accuracy on edge devices remains a trade-off. Additionally, many ML models act as “black boxes,” limiting interpretability. However, techniques like Grad-CAM and t-SNE are beginning to provide insights into model decision-making, increasing trust (Jadesha et al., 2026; Licaj et al., 2023). Data scarcity and class imbalance are also recurrent issues, often mitigated by augmentation or synthetic data, but the need for large. Addressing these requires multidisciplinary collaboration among breeders, data scientists, and policy makers to foster user-friendly AI tools that integrate seamlessly into breeding workflows and engage the farming community (Mushtaq et al., 2024).

AI and metadata integration form a cornerstone of modern breeding programs for root, tuber, and cereal crops, driving unprecedented gains in selection precision and breeding efficiency. Future progress will depend on developing robust, explainable, and computationally efficient models that are validated on diverse field datasets and integrated into accessible decision-support tools. Its strategic deployment is pivotal for developing climate-smart, high-yielding, and nutritionally superior cultivars needed to meet global food security challenges in a changing environment (Zhu et al., 2025; Mushtaq et al., 2024). Fostering collaborations between data scientists, breeders, and agronomists is essential to translate these technological advances into tangible gains in crop productivity, resource efficiency, and climate resilience for global food security. For tasks requiring real-time, on-device deployment, lightweight models like YOLOv8n and Random Forest offer the best balance of accuracy and efficiency. When predicting complex biological traits (e.g., yield, growth rate) from tabular environmental data, k-Nearest Neighbors (kNN) and Artificial Neural Networks (ANN) often outperform other methods, though ensemble approaches generally provide the most robust and generalizable solutions across diverse conditions. **Tables 2** and **3** summarize AI and the deep learning applications in crop breeding.

### 3.3 Application of pangenomics, multi-omics integration and systems biology approaches in breeding

Root, tuber, and cereal crop improvement via genomics faces key challenges, complex polyploidy and high heterozygosity (potato, wheat); large, repetitive genomes that hinder gene mapping; and limited genetic diversity due to domestication bottlenecks and selective breeding. Precise trait dissection is difficult for polygenic stress responses (drought, heat, salinity, disease) requiring integration of multi-omics and high-throughput phenotyping. Many beneficial alleles in wild relatives remain underutilized due to barriers in introgression and incomplete functional annotation. Scaling genome sequencing, resolving presence–absence and structural variation, and linking genotype to phenotype are critical for breeding resilient, high-yielding varieties in these globally important crops (Tiwari et al., 2022; Hussain et al., 2022).

Pangenomics and systems biology have become pivotal in accelerating crop improvement, particularly for cereals and root/tuber crops. Pangenomes, constructed by sequencing multiple accessions, capture a species’ full genetic diversity—including core and dispensable genes—extending beyond what single reference genomes can reveal (Danilevicz et al., 2020; Varshney et al., 2006; Yang et al., 2021; Zanini et al., 2022; Ikan et al., 2025). In crops like rice, maize, wheat, barley, and sorghum, pangenome studies have discovered novel genes linked to stress adaptation, disease resistance, and yield. These resources facilitate the recovery of beneficial genes lost during domestication and support targeted introgression from wild relatives to bolster climate resilience and productivity (Yang et al., 2021; Sato, 2025; Guo et al., 2025a, b).

Systems biology enables the integration of multi-omics data—genomics, transcriptomics, proteomics, and metabolomics—using computational models to unravel complex trait architectures (Kumar et al., 2015; Kumar et al., 2022; Yang et al., 2021; Ikan et al., 2025). Approaches vary from top-down (mining omics datasets for regulatory elements) to bottom-up (simulating gene networks to predict phenotypic outcomes), guiding the identification of promising targets for breeding and predicting gene function under different environments (Weisweiler et al., 2019; Sow et al., 2025).

Combining pangenomics with systems biology creates a powerful framework for linking genetic variation to phenotype. This synergy accelerates discovery of causal loci, regulatory modules, and adaptive alleles, making genomic selection and precision breeding more effective (Yang et al., 2021; Tay Fernandez et al., 2022). For instance, a study on barley using multi-tissue mRNA-Seq revealed thousands of genes missed by traditional references, while integration of presence/absence and expression variation improved prediction of agronomic traits. Such findings are directly transferable to polyploid cereals and complex-root or tuber crops (Weisweiler et al., 2019).

Wheat pangenomic resources have enabled mapping of key adaptive genes and gene families involved in stress resistance, quality, and fertility, thanks to high-quality, chromosome-scale assemblies (IWGSC International Wheat Genome Sequencing Consortium, 2018; Walkowiak et al., 2020; Hussain et al., 2022; Tiwari et al., 2024). Integrating long-read and Hi-C assemblies has revealed introgressions, centromere shifts, and novel gene family expansions, powering trait mapping and supporting CRISPR-based breeding. These approaches are mirrored in barley, where graph-based pangenomes and multi-omics guide the use of gene bank diversity and accelerate marker-assisted selection (Jayakodi et al., 2024; Sato, 2025).

Potato pangenomes, generated from phased, long-read sequencing of diverse historical and wild-accession genomes, have captured most haplotype diversity and revealed extensive introgression and selection. Genome graphs now enable cost-effective genotype imputation and accelerate marker discovery and gene editing (Sun et al., 2025; Cheng et al., 2025a). Similarly, cassava’s pangenome has resolved domestication signals and key gene family variation, linked directly to traits like cyanogenic potential and tuber formation (Xia et al., 2025).

In cereals, studies like those of Sow et al. (2025) demonstrate “inter-crop translational breeding”, genes and selective sweeps identified in wheat-barley comparative genomics are used to inform selection in other crops, even those with differing ploidy. These methods also reveal unique alleles in ancient lineages for climate resilience.

For sorghum, genome–environment association mapping (Lasky et al., 2015) and integration of genome-wide SNPs with environmental and phenotypic data predict adaptive outcomes and guide the prioritization of loci for functional study, illustrating the predictive power of systems-level multi-omics, especially when using a pangenome as the foundation.

Smart breeding strategies now employ high-throughput sequencing, machine learning (ML), and digital phenotyping to leverage pangenomic information for climate adaptation, disease prediction, and yield optimization (Naqvi et al., 2022). ML, combined with genomic selection and CRISPR editing, enables breeders to mine and utilize useful alleles more efficiently and to predict trait outcomes more accurately.

Conventional approaches, while vital, have been relatively slow and rely on narrow genetic pools. Modern genomics underpinned by pangenome and multi-omics strategies is revolutionizing improvement in both major cereals and underutilized crops, by enabling efficient capture of diversity, faster breeding cycles, precise marker and ideotype discovery, and a clear roadmap for resilient crop development (Yang et al., 2021; Tiwari et al., 2022; Aziz and Masmoudi, 2025; Montenegro et al., 2017).

Recent advances in crop genomics demonstrate that integrating diverse data types significantly enhances breeding efficiency. Structural variants (SVs) and presence-absence variations (PAVs) often underlie major-effect traits. In rice, a PAV at the Se locus controls hybrid sterility via a killer-protector system (Wang et al., 2023). Similarly, in sweet potato, a PAV in IbMYB1–2 explains over 75% of flesh color variation (Zhang et al., 2020). These findings highlight that SVs and PAVs are critical for reproductive isolation and quality traits. Standard SNP markers remain essential for complex traits. High-density SNP panels enabled genomic selection (GS) in rice breeding lines (Chaity et al., 2026). In yam, 4,525 SNPs identified stable markers for tuber yield and virus resistance (Adewumi et al., 2024). Cassava protein content was improved using 8.17 million SNPs to identify major-effect loci (Wang et al., 2025b). Thus, both structural and sequence variations are vital for comprehensive genetic dissection. Cheng et al. (2025b) constructed a graph pangenome from 22 hexaploid wheat assemblies, identifying ~1.98 million non-redundant SVs and 497 SV hotspots, with notable aggregation in centromeric regions. This resource reveals untapped genetic diversity and improves genome-wide association mapping by linking SVs to agronomic traits like growth habit. Shi et al. (2020) integrated metabolomics with agronomic traits in wheat kernels, detecting 1,260 metabolic features and 1,005 mQTLs. Their work identified candidate genes modulating flavonoids and auxin accumulation, demonstrating that metabolite profiles can predict traits such as grain number per spike and plant height. Liu et al. (2025b) incorporated transcriptome data into genomic selection models for flowering time and height, showing that combining SNP, transcript, and environmental data enhances prediction accuracy, especially under controlled conditions. Jiang et al. (2025a) developed a Wheat Quality Molecular Marker Selection System (QMMS) using KASP markers for key quality loci, enabling cost-effective, high-throughput selection for grain protein, hardness, and glutenin subunits. In barley, Jiang et al. (2025b) outlined a multi-omics vision leveraging SVs, expression, and metabolite data to accelerate breeding for yield, resilience, and quality.

Metabolomics provides a direct link between genotype and nutritional quality. In cassava, profiling 2,980 metabolites revealed genes controlling cyanogenic glucosides and anthocyanins (Ding et al., 2023). Maize studies identified 3,020 metabolites linked to flavor and lipid content (Li et al., 2025a). Carrot metabolomics identified 607 compounds associated with sweetness and bitterness (Gao et al., 2025). These metabolite profiles enable selection for hard-to-phenotype traits. Transcriptomics further elucidates regulatory mechanisms. RNA-Seq in cassava identified defense genes for root rot resistance (Hohenfeld et al., 2024). Expression quantitative trait locus (eQTL) mapping in sweet potato revealed regulatory networks for anthocyanin biosynthesis (Zhang et al., 2020). Carrot transcriptomics highlighted stage-specific gene expression during taproot maturation (Gao et al., 2025). Bolting resistance in carrot was linked to specific gene expression patterns (Kong et al., 2025). Integrating expression data with genomics clarifies gene function and regulation.

Breeding decisions are directly enabled by these multi-omics insights. Marker-assisted selection (MAS) utilizes diagnostic SNPs and PAVs. Rice breeders can screen for compatible Se haplotypes to overcome hybrid sterility (Wang et al., 2023). Cassava breeders can select for low linamarin using validated SNPs (Ding et al., 2023). Yam breeders can pyramid alleles for yield and virus resistance (Adewumi et al., 2024). Candidate genes provide targets for genome editing. Rice ORF3 and ORF4 can be edited to create neutral alleles (Wang et al., 2023). Cassava MeFLS1 and MeMYB4 are targets for yield and color improvement (Ding et al., 2023). Maize ZmGPAT11 editing can enhance lipid content (Li et al., 2025a). Carrot LOC108205243 offers a target for bolting control (Kong et al., 2025). These validated genes accelerate precise genetic modification.

Genomic selection (GS) models benefit from multi-omics data integration. Rice breeding programs combine MGIDI indices with GS to accelerate genetic gain (Chaity et al., 2026). Cassava GS models incorporate metabolite associations to predict nutritional quality (Ding et al., 2023). Maize GS models achieve high accuracy using metabolome-informed markers (Li et al., 2025b). Databases like EMMDB facilitate precision breeding in maize (Li et al., 2025b). High-protein cassava lines were identified using GWAS and heritability estimates (Wang et al., 2025c). These models reduce breeding cycles and improve selection accuracy. Polyploid crops require specialized analytical approaches. Sweet potato eQTL mapping accounted for hexaploid complexity (Zhang et al., 2020). Yam GWAS utilized specific gene action models for tetraploid species (Adewumi et al., 2024). Addressing ploidy levels ensures accurate marker detection.

**Figure 2:**
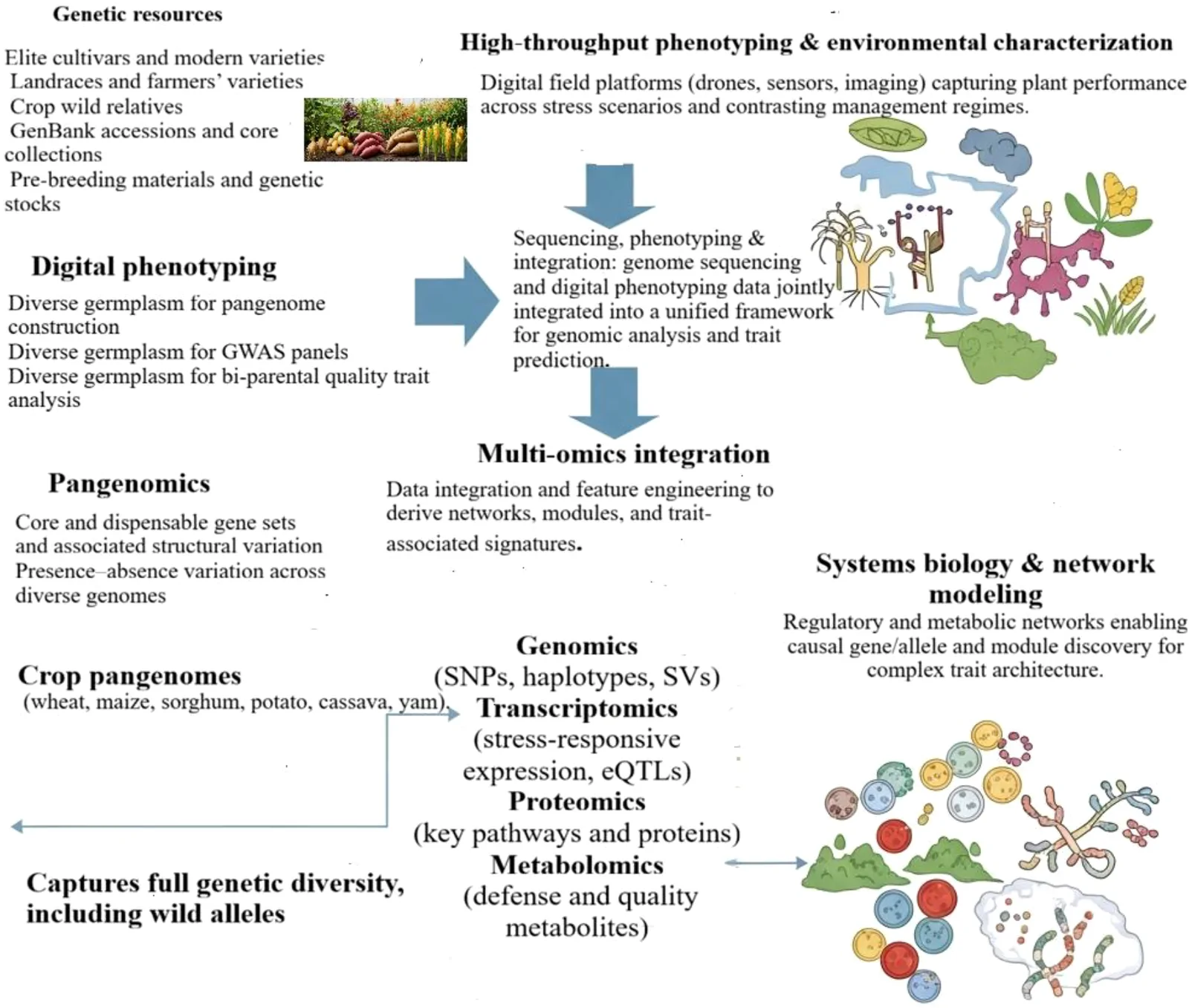
Genomics-enabled breeding landscape for root, tuber, and cereal crops.

Collectively, these advances not only deepen biological insight but also provide pragmatic tools for addressing global food security and environmental challenges in an era of rapid climate change (Yang et al., 2021). The applications of pangenome, multi-omics, and systems biology in crop breeding are presented in **Table 4**. The road map for the genomics-enabled breeding landscape for root, tuber, and cereal crops is presented in **Figure 2**.

**Table 4:** Pangenome, multi-omics, and systems biology applications in crop breeding.

Crop	Approach/method	Major findings	Relevance to breeding	References
Maize	Genetic mapping of 26 million GBS tags; pan-genome characterization; integration of 46 genomes with RNA-seq and SV visualization; pan-genomics + RNA-seq on diverse lines	4.4M pan-genome anchors incl. 1.1M PAVs linked to agronomic traits - Extensive PAV and structural variation; transcript diversity linked to heterosis	Enables precision breeding by linking structural variation and novel transcripts to traits Tools to compare gene content and expression across lines	Lu et al., 2015; Woodhouse et al., 2021; Hirsch et al., 2014
Rice	Near-isogenic lines + map-based cloning; CRISPR/Cas9; population genomics (787 accessions); cytology, qRT-PCR	PAV at Se locus causes hybrid male sterility via killer-protector system (ORF3/ORF4) Functional ORF3 enriched in indica; most japonica lack PAV	Se is major target to overcome indica-japonica reproductive isolation Neutral alleles via Type-3 Se mining or ORF3 editing Linked markers enable MAS for compatibility	Wang et al., 2023
	Multi-environment phenotyping (216 F_7_ lines × 2 seasons); MGIDI index; Genomic selection using 995 SNPs (GBS)	Significant genetic variability for yield (GCV = 14–18%, h²=57–74%) MGIDI identified IR19A8066, IR19A8052 as top multi-trait genotypes 100/200 lines showed positive GEBVs (0 to 0.68)	MGIDI overcomes multicollinearity limitations for simultaneous multi-trait selection GEBV-based parental selection accelerates genetic gain and shortens breeding cycles	Chaity et al., 2026; Guo et al., 2025a
	145 chromosome-level assemblies; gene and graph-based pangenomes; population genomics and phylogenomics	- 69,531 pan-genes, 13,728 wild-specific genes - Wild rice as reservoir of resistance alleles - Domestication and divergence SNPs and PAVs identified	- Supports introgression of stress resistance alleles from wild relatives - Enables precision breeding to avoid linkage drag	Guo et al., 2025a
Sorghum	Genome-environment association (GEA), GWAS	Environmental SNPs predict adaptation and yield; identified candidate adaptation genes	Enables predictive breeding and identification of adaptive genes for stress tolerance	Lasky et al., 2015
Wheat	Pangenome assembly, comparative genomics	Revealed PAVs, CNVs, introgressed genes, chromosome assemblies	Expands genetic diversity; supports trait mapping, marker development, and hybrid breeding	Walkowiak et al., 2020
Barley	Long-read pangenome, multi-omics, graph-based trait mapping	Captures large-scale structural variation; functional loci	Enables resistance discovery, trait mapping, and enhanced genomic selection	Jayakodi et al., 2024
Potato	Phased/graph-based pangenome with long reads and Hi-C, multi-omics	Detailed haplotype and allele mining, introgression detection; deleterious variant tracking	Accelerates marker discovery, haplotype design, and genomic selection	Sun et al., 2025; Cheng et al., 2025b
Cassava	Haplotype-resolved T2T and pangenome with multi-omics	Defined core/dispensable genes, linked domestication traits; causal variant validation	Framework for targeted introgression, gene editing, and marker development	Xia et al., 2025; da Costa et al., 2024
Yam	Multi-omics, Genome-environment association (GEA), GWAS	Detailed haplotype and allele mining, Environmental SNPs predict adaptation and yield; identified candidate adaptation genes	Accelerates marker discovery, haplotype design, and genomic selection; Enables predictive breeding and identification of adaptive genes for stress tolerance	Chen et al., 2024; Agre et al., 2021; Darkwa et al., 2020, Darkwa et al., 2019; Gatarira et al., 2020
Carrot	Widely targeted metabolomics (UPLC-MS/MS), 607 metabolites across 3 developmental stages; RNA-Seq transcriptomics	607 metabolites in 14 categories; lipids (21.7%) and phenolic acids (15.1%) most abundant Sugar accumulation (fructose, glucose) primarily occurs S→L stage (30–50 DAS)	Candidate genes for quality improvement, SPS/SUS/INV (sugar balance), HCT (chlorogenic acid biosynthesis) Metabolic markers for MAS, fructose/glucose ratio (sweetness), chlorogenic acid content	Gao et al., 2025
	Multi-environment phenotyping (240 accessions × 4 locations × 2 years); Whole-genome resequencing (~10× depth); GWAS via MLM	19.1M high-quality SNPs; LD decay at 35 kb (r²=0.1) 9 SNPs significantly associated with bolting speed; peak SNP at Chr1:33,254,067 93 candidate genes within ±100 kb; LOC108205243 (E3 ubiquitin ligase) prioritized	Peak SNP enables development of KASP/CAPS markers for bolting tolerance LOC108205243 provides target for gene editing to modulate bolting timing 71 tolerant accessions serve as immediate donor parents	Kong et al., 2025
Cassava	Multi-time-point phenotyping (0–60 DAI) of resistant vs. susceptible genotypes; RNA-Seq transcriptomics (20 libraries)	23,912 DEGs identified; resistant genotype shows constitutive enrichment of redox; NTP-binding GO terms Metabolic reprogramming, early upregulation of carbohydrate/nucleotide metabolism; late activation of lignin biosynthesis, ROS, JA/BR signaling	Validated candidate genes enable development of functional molecular markers for MAS of root rot resistance • BRS Kiriris serves as elite donor parent for introgression of quantitative resistance	Hohenfeld et al., 2024
	Widely targeted metabolomics (UPLC-MS/MS), 2,980 metabolites in storage roots of 299 cultivated accessions; Whole-genome resequencing, 1.16M high-confidence SNPs; mGWAS + pGWAS	489 annotated metabolites; 74% H² > 0.5; 64.3% CV > 50% 18,218 significant marker-metabolite associations; 158 mGWAS hotspots Me3GT, causal SNP (P334S) controls kaempferol glucosylation; CG cluster (CYP79D1/CYP71E7b/UGT85K5) reduces linamarin	Validated causative SNPs enable functional marker development (KASP/CAPS) for MAS of nutritional traits, food safety, and yield • MeFLS1 provides dual-target opportunity, edit promoter to reduce quercetin 3-O-glucoside and boost yield	Ding et al., 2023
	Multi-year phenotyping (261 F_1_ lines × 3 years) for storage root protein content; Whole-genome resequencing (20× depth); GWAS via MLM	Protein content, range 1.01–7.12%, mean 3.05%, CV 13%, H² = 0.68 21 significant SNPs on 13 chromosomes; SNP_6831776 (Chr15) and SNP_7090537 (Chr16) with R² = 0.35 82 candidate genes; 6 of 7 tested genes upregulated in high-protein lines	Major-effect SNPs enable marker-assisted selection for rapid protein content improvement • Validated candidate genes provide targets for functional marker development and potential gene editing • 21 elite high-protein lines serve as immediate donor parents	Wang et al., 2025c
Sweet potato	Transcriptome sequencing (88 accessions) + resequencing (16 accessions); eQTL mapping via EMMAX; GWAS for flesh color; PAV detection for IbMYB1-2	724,438 high-confidence SNPs identified in hexaploid genome 4,408 eQTLs regulating 3,646 genes (2,261 local, 2,147 distant) IbMYB1–2 identified as master regulator, controls 17+ flavonoid biosynthesis genes; PAV explains 75.65% of flesh color variation	IbMYB1–2 PAV markers enable rapid MAS for purple-fleshed sweet potato breeding Flavonoid regulatory network provides targets for metabolic engineering of anthocyanin content 724K SNP resource supports genomic selection for complex, polygenic traits in hexaploid sweet potato	Zhang et al., 2020
Yam)	Multi-environment phenotyping (132 accessions × 2 seasons); DArTseq genotyping (4,525 SNPs); GWAS via MLM with four gene action models	27 significant SNPs across 13 chromosomes; 5 stables across seasons (3 for TWPL, 2 for YMV) Heritability, TWPL (60%), YMV (99%), TSR (46%) 10 candidate genes for tuber yield, protein kinases, LRR domains, BRX transcription factors	Stable SNPs enable marker-assisted selection for tuber yield and YMV resistance across environments Validated candidate genes provide targets for functional marker development and potential gene editing Haplotype information informs crossing strategies to pyramid favorable alleles	Adewumi et al., 2024
Edible Maize	Whole-genome resequencing (452 accessions, 9.42× depth); Widely targeted metabolomics (LC-MS/MS), 3,020 metabolites, 802 annotated; mGWAS + pGWAS	Three subgroups (sweet, waxy, field) show distinct metabolic profiles (FST, 0.028–0.079) 6,313 mGWAS signals for 802 metabolites; 158 association hotspots Convergent metabolic differentiation, flavonoids decreased (reduced bitterness), lipids increased (enhanced nutrition)	Validated causative SNPs enable functional marker development for MAS of flavor and nutritional traits ZmGPAT11 and flavonoid pathway genes provide targets for metabolic engineering of kernel quality EMMDB database supports precision breeding via metabolite prediction and parental selection	Li et al., 2025

GBS, genotyping−by−sequencing; PAV, presence–absence variation; SV, structural variation; RNA−seq, RNA sequencing; GCV, genotypic coefficient of variation; h², narrow−sense heritability; MGIDI, multi−trait genotype–ideotype distance index; MAS, marker−assisted selection; GEBV, genomic estimated breeding value; F_7_, seventh generation of selfed lines; CNV, copy−number variation; GEA, genome−environment association; GWAS, genome−wide association study; MLM, mixed linear model; UPLC−MS/MS, ultra−performance liquid chromatography–electrospray ionization–tandem mass spectrometry; DEGs, differentially expressed genes; GO, gene ontology; JA, jasmonic acid; BR, brassinosteroid; mGWAS, metabolome−wide association study; pGWAS, pan−genome−wide association study; HCT, hydroxycinnamoyl−transferase; H², broad−sense heritability; CV, coefficient of variation; qRT−PCR, quantitative real−time polymerase chain reaction; NTP, nucleoside triphosphate; ROS, reactive oxygen species; TWPL, tuber weight per plant; YMV, yam mosaic virus; TSR, tuber starch ratio; DArTseq, diversity arrays technology by sequencing; EMMAX, efficient mixed−model association expedited; LRR, leucine−rich repeat; BRX, BRX−domain transcription factor; TWPL, tuber weight per plant; DAI, days after inoculation; DAS, days after sowing; 2D, two−dimensional; LC−MS/MS, liquid chromatography–tandem mass spectrometry; EMMDB, edible maize metabolome database; ZmGPAT11, maize glycerol−3−phosphate acyltransferase 11; KASP, kompetitive allele−specific PCR; CAPS, cleaved amplified polymorphic sequence; eQTL, expression quantitative trait locus; IbMYB1−2, Ipomoea batatas MYB transcription factor 1−2; mGWAS hotspot, genomic region with a high density of metabolite–marker associations; pGWAS hotspot, genomic region with a high density of pan−genome−based marker–metabolite associations. .

## 4 Prospects of artificial intelligence, pangenomics, multi-omics, precision agriculture, and personalized breeding

### 4.1 Status and prospects of pangenomics technologies

Pangenomics captures structural variants and rare alleles missed by single references, while multi omics links regulatory and metabolic layers to phenotypes. Recent AI models (e.g., graph aware GS, multi modal DL) leverage these datasets to improve trait prediction and identify candidate genes for editing and introgression in maize, rice, wheat, potato, and cassava. Pangenomics technologies have advanced significantly due to improvements in sequencing, computational methods, and integration of artificial intelligence (AI). Unlike traditional single reference genomes, pangenomics captures the entire genetic diversity within a species by assembling genomes from multiple individuals. This approach better represents structural variations and gene presence-absence variants, which are key to understanding complex traits. The use of graph-based pangenome models allows efficient representation of genomic diversity and precise identification of genetic variants at the base-pair level (Hu et al., 2024; Wang et al., 2025b). Recent developments in long-read sequencing technologies such as HiFi sequencing have enabled the construction of more accurate and comprehensive pangenomes. Computational pipelines like Minigraph-Cactus support effective pangenome assembly by handling large-scale structural variations with increasing speed and accuracy (He et al., 2025; Li et al., 2022). Applications of pangenomics span major and orphan crops like rice, maize, sorghum, and tomato. This allows breeders and researchers to detect genetic variants linked to stress resistance, yield, or quality traits that are missed by single reference genomes (Hu et al., 2024; Wang et al., 2025b). Looking forward, the integration of AI and machine learning with pangenomic data is transforming plant breeding through enhanced variant discovery and genotype-to-phenotype prediction, particularly for traits related to climate change adaptation (He et al., 2025; Amin et al., 2025). Furthermore, multi-omics approaches that combine pangenomics with transcriptomics, proteomics, and metabolomics are being developed to provide a holistic view of trait regulation and improve precision breeding strategies (Amin et al., 2025). Despite these advances, challenges such as high costs, computational demands, and equitable data sharing remain. Large collaborative efforts like the Earth BioGenome Project facilitate open-access data sharing to address these limitations and accelerate global research applications for biodiversity conservation and crop improvement (He et al., 2025). Thus, pangenomics is at the forefront of genomics research, with promising prospects driven by evolving sequencing technologies, AI integration, and international collaborations aimed at enhancing crop resilience and agricultural productivity.

### 4.2 Status and prospects of multi-omics technologies

**Figure 3:**
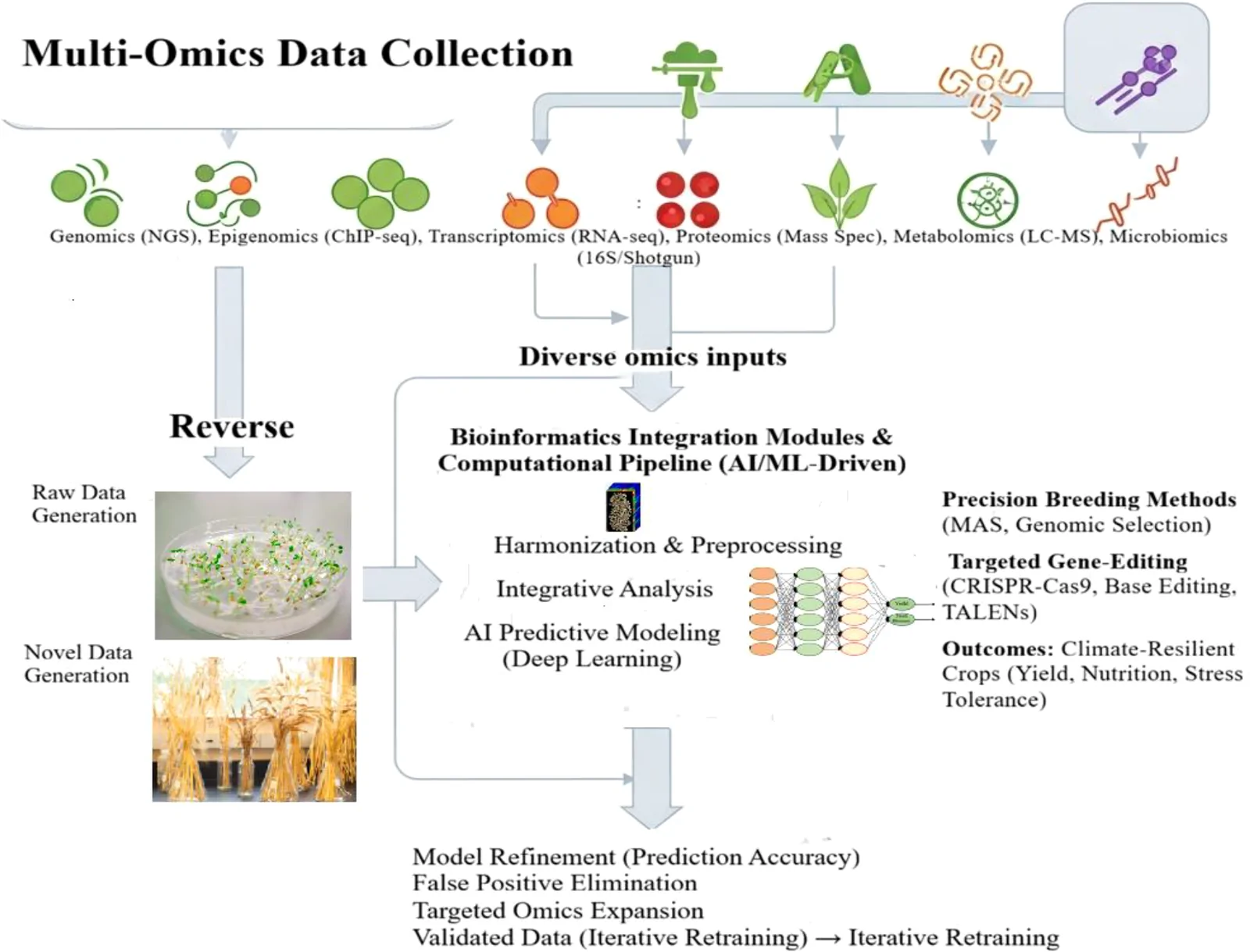
Integrated multi-omics pipeline for precision crop improvement.

Multi-omics technologies, including genomics, transcriptomics, proteomics, metabolomics, and microbiomics, have revolutionized agricultural research by offering a holistic view of plant biology and stress responses. These integrated approaches enable the identification of crucial genes, proteins, and metabolites that drive growth, yield, and resilience against environmental stresses, which are intensified by climate change challenges (Younas et al., 2025). By combining multi-omics with artificial intelligence and genome editing, precision breeding is accelerated to produce climate-resilient, high-yielding, and nutritionally improved crops (Zhang et al., 2022). These methods elucidate complex genotype-phenotype relationships and regulatory networks, thereby enhancing the accuracy and efficiency of breeding programs for sustainable agricultural practices (Kumar et al., 2025). Although challenges such as data heterogeneity, cost, and computational complexity remain, advances in high-throughput sequencing, mass spectrometry, and AI analytics are progressively overcoming these barriers (Dong et al., 2024). Emerging directions emphasize real-time multi-omics analysis, multi-stress modeling, and extension to underrepresented crops, promising profound improvements in breeding efficiency and global food security (Sarfraz et al., 2025; Kajrolkar, 2025). Integration of multiple omics layers has thus become essential for decoding the molecular mechanisms underlying plant stress tolerance and for designing next-generation crops that meet agricultural demands amidst environmental uncertainties (Younas et al., 2025; Kumar et al., 2025; Zhang et al., 2022). For instance, integrated transcriptome-metabolome studies under combinatorial stresses have uncovered tissue-specific regulatory networks, in wheat, flavonoid and phenolic acid biosynthesis distinguished drought-tolerant from susceptible EMS mutants (Guo et al., 2025b); in barley, ROS scavenging and β-glucosidase activity underpinned malting quality stability in wild accession XZ166 (Hong et al., 2020); and in maize, cytokinin deficit via downregulated ZmIPT6/8 and sucrose synthase suppression explained reproductive abortion under heat × drought (Li et al., 2025). To ensure robust multi-omics inference, a concise design checklist is critical, maintain adequate biological replicates (n≥3–6), apply blocking and randomization to control environmental heterogeneity, and implement batch correction during data preprocessing. These methodological safeguards enhance reproducibility and enable precise identification of candidate genes and metabolites for marker-assisted selection. Collectively, such integrated frameworks accelerate the development of climate-resilient cultivars by linking dynamic molecular responses to agronomic performance under complex stress scenarios. **Figure 3** presents the integrated multi-omics pipeline for precision crop improvement.

## 5 Advances in fluorescence-based hybridization and microscopic imaging technologies

**Figure 4:**
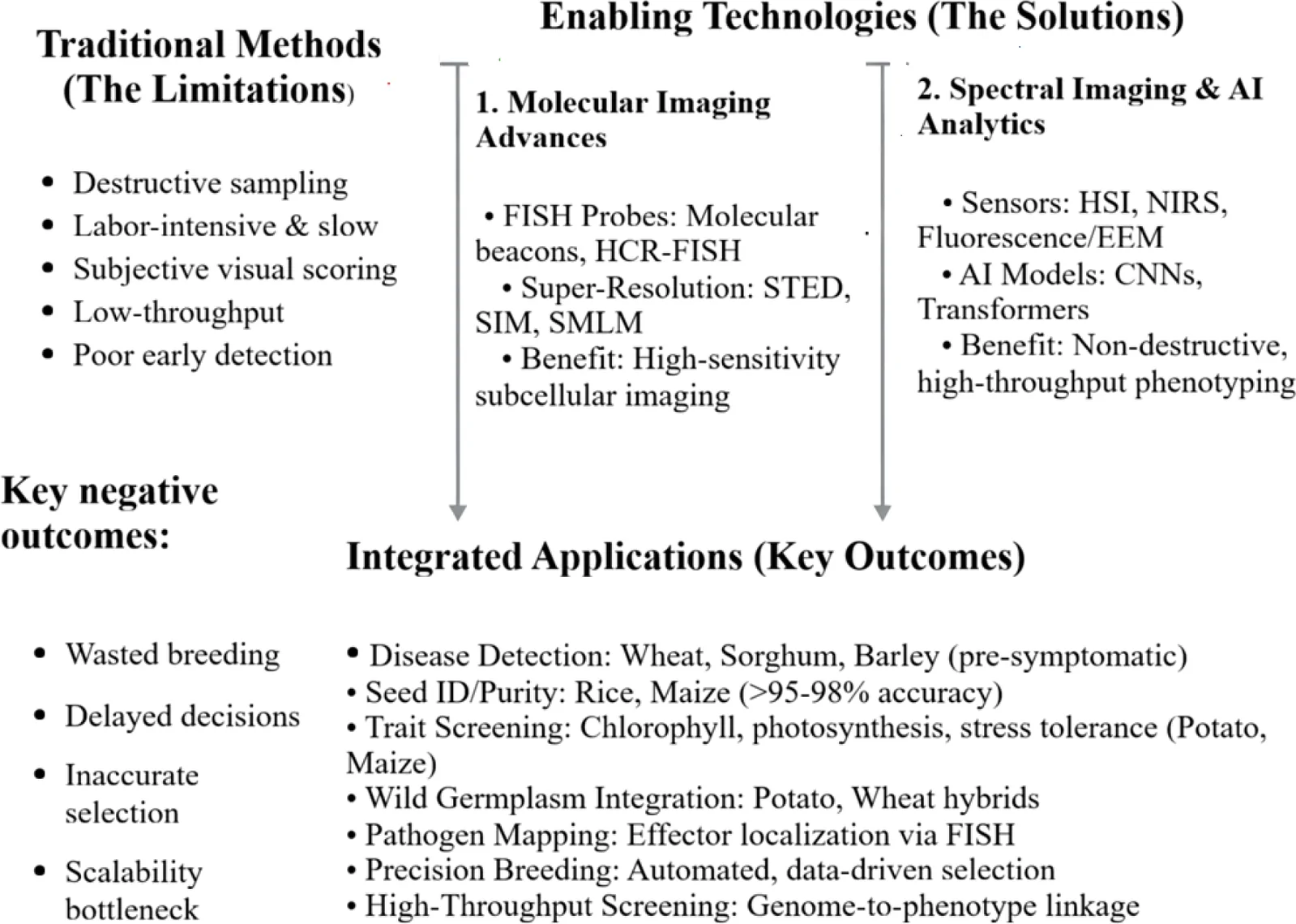
Fluorescence-based hybridization and advanced imaging.

Traditional methods for seed and grain quality assessment and plant disease diagnosis are often destructive, labor-intensive, and slow. They require complex sample preparation, rely on subjective visual scoring or time-consuming chemical assays, and typically analyze only surface or single-point traits, missing deeper or internal variation. These approaches are poorly suited for high-throughput or real-time applications and cannot detect early or asymptomatic infections. Their accuracy is limited by human error and environmental factors, and they lack compatibility with automated breeding or industrial workflows. As a result, they hinder the speed, scale, and precision needed for modern crop improvement and risk management (Liang et al., 2025). Fluorescence-based hybridization and advanced imaging (**Figure 4**) have driven major breakthroughs in root, tuber, and cereal crop breeding. Recent improvements in fluorescence *in situ* hybridization (FISH), notably with molecular beacon probes and signal amplification methods like HCR, SABER, and FRET-based beacon probes, have boosted both sensitivity and multiplexing, allowing detailed and low-noise tracking of nucleic acids and cellular targets (Choijookhuu et al., 2022; Morales et al., 2022). These advances enable rapid detection of pathogens and facilitate the integration of wild germplasm into breeding programs even in genetically complex crops like potato and wheat, which rely on the unique resolution offered by these techniques for mapping and introgression (Gaiero et al., 2018; Matveeva et al., 2024).

Microscopy technologies have simultaneously leaped forward. Super-resolution approaches like STED, SIM, and single-molecule localization microscopy have overcome the diffraction limit, enabling detailed study of subcellular plant-pathogen interactions. Deep learning-based image enhancement increases throughput and clarity, from photon to X-ray and electron imaging, offering ultrafast and deep 3D insights (Nkune et al., 2025; Dutta et al., 2024; Zhou et al., 2023). These tools, complemented by light sheet and confocal platforms, uncover microbial colonization, effector localization, and host responses across spatial and temporal scales in living tissues—crucial for understanding disease progression and resistance in crops (Hilaili et al., 2026).

In seed and grain phenotyping, non-destructive, AI-enhanced spectral imaging is replacing traditional, laborious assays. Near-Infrared Spectroscopy (NIRS) and Hyperspectral Imaging (HSI) have enabled rapid, high-accuracy screening of seed variety, purity, and composition, powering advances in both rice (Jin et al., 2022; Zhang et al., 2025a) and maize (Xu et al., 2026) by using deep learning models to classify varieties and monitor grain quality. Innovative approaches like CNN-MFF, transformer networks, and spectrum fusion achieve varietal identification rates above 95%, aiding seed certification and genotyping (Zhang et al., 2025b; Jin et al., 2022; Xu et al., 2026).

Likewise, fluorescence and EEM spectroscopy, paired with multivariate analysis, have empowered early, non-invasive disease detection in wheat and sorghum, even pre-symptomatically, accelerating resistance breeding (Matveeva et al., 2024; Hilaili et al., 2026). In potato and maize, hyperspectral reflectance and advanced imaging models permit fast, robust assessment of photosynthetic efficiency and chlorophyll content (Zhao et al., 2021; Guohui et al., 2025), traits linked to yield and stress tolerance.

Despite advances in fluorescence-based hybridization and microscopic imaging technologies for agricultural phenotyping, several general limitations hinder widespread adoption. Throughput and cost remain critical barriers; high-resolution systems often entail expensive hardware and computational complexity that preclude real-time, edge-based deployment in resource-constrained environments. Standardized spectral databases, interpretable AI models, and integrated pipelines are needed for reliable deployment (Mohammadi-Shemirani et al., 2023; Liang et al., 2025). Instrument variability necessitates robust calibration transfer protocols to ensure model portability across different sensors, as device-specific spectral responses often limit interoperability. Furthermore, a significant field-to-lab domain shift undermines robustness, where models trained under controlled conditions fail to generalize to dynamic field environments due to uncontrolled ambient light, dust, vibration, and environmental heterogeneity. Finally, reliance on small sample sizes, limited varietal diversity, and single-location data restricts model generalizability, highlighting the need for multi-environment validation and standardized protocols to bridge the gap between laboratory proof-of-concept and scalable field applications. **Table 5** presents summaries of advanced fluorescence and spectral imaging applications in selected crops.

**Table 5:** Overview of advanced fluorescence and spectral imaging applications in major crop breeding.

Crop	Main technology	Core method/principle	Major breeding/screening benefit	Limitations	Reference
Sorghum	Fluorescence spectroscopy	EEM + imaging under specific UV/visible	Rapid, non-destructive screening of grain quality	Include the focus on discrete milling endpoints, limited varietal scope, instrument-specific calibration requirements, unvalidated industrial throughput and cost, and field-to-lab domain shifts that may compromise real-world robustness.	Hilaili et al., 2026
Wheat	Fluorescence spectroscopy	EEM + PCA	Early, non-invasive detection of fungal disease	Laboratory-scale EEM fluorescence detection of wheat pathogens is limited by manual inoculation, instrument complexity, unvalidated field robustness, and inferential metabolite attribution, hindering rapid in-line grain screening deployment.	Matveeva et al., 2024
Barley	Hyperspectral phenotyping	Multispectral imaging + microscopy + PCA	Early detection of stress/disease resilience traits	Model-to-crop translation gaps, scarce field validation of predictive models, high computational/expertise barriers, and limited applicability to resource-constrained agricultural systems.	Pandey et al., 2025
Rice	HSI and deep learning	CNN-Transformer with feature selection	Fast, accurate grain varietal ID for breeding/quality	Hybrid indica-only scope, single-location/single-season sampling, unvalidated field robustness, and computational demands hindering real-time	Zhang et al., 2025b
	NIR HSI and deep learning	ResNet CNN + saliency maps	Automated seed variety/purity screening	Varietal scope, single-location sampling, small per-variety sample sizes, 1D spectral-only analysis, and unvalidated field robustness and computational efficiency for real-time industrial deployment.	Jin et al., 2022
	HSI and deep transfer learning	1D-CNN, fine-tuning, transfer learning	Scalable cross-variety disease phenotyping	Small sample sizes, laboratory greenhouse conditions, and lack of validation across different regions, equipment, or standardized spectral databases for robust disease detection applicability in field scenarios	Feng et al., 2021
Maize	Fluorescence imaging and deep learning	BLF-CLAHE corrected MSI, CNN	Robust, non-destructive chlorophyll content screening	Single-variety maize data from one location, device-specific fluorescence calibration requirements, and unvalidated generalizability across diverse environmental conditions, crop varieties, or imaging equipment for robust field-scale chlorophyll diagnosis.	Guohui et al., 2025
	HSI and deep learning	CNN-MFF, spectral fusion, feature selection	High-throughput, accurate seed variety certification	Small sample size, single-location/single-season data, unvalidated cross-device transferability, impractical bilateral spectral fusion requirement, and computational complexity hindering real-time edge deployment for field-scale seed quality control	Xu et al., 2026
Potato	Hyperspectral spectral analysis	HSI, CWT, RF, PLS regression	High-throughput photosynthetic efficiency selection	Tuber-formation-stage sampling, laboratory-only validation, and marginal model robustness for field canopy monitoring.	Zhao et al., 2021
Cassava	NIR HSI and deep learning	ResNet CNN + saliency maps	Automated variety/purity screening	Cassava and yam calibration datasets, inconsistent spectral protocols across studies, poor instrument interoperability, underexplored end-product quality traits, and insufficient validation for field-deployable breeding applications.	Alamu et al., 2021
Yam					

## 6 Environmental adaptability and breeding strategies under climate change

Environmental adaptability in crop breeding under climate change has become a critical focus of contemporary agricultural research and practice. Climate change introduces multiple abiotic stresses, including drought, heat, flooding, and salinity, which impair crop productivity and threaten food security globally. Breeding strategies now prioritize developing climate-resilient crop varieties that can endure these stresses while maintaining or improving yield. Modern breeding approaches leverage the full spectrum of genetic diversity available within and across species, often incorporating wild relatives and landraces as reservoirs of adaptive traits. Adaptation is accelerated through advanced genomic-assisted breeding techniques, including genome editing tools such as CRISPR and marker-assisted selection. These enable rapid identification and incorporation of favorable alleles related to stress tolerance, efficient water and nutrient use, and disease resistance. Crop phenotyping, combined with envirotyping—detailed environmental characterization—is integrated to better understand genotype-by-environment interactions, which are vital for selecting resilient genotypes. Complementary agronomic practices, such as crop rotation and integrated pest management, support the overall resilience of agroecosystems.

Next-generation breeding platforms embody the integration of multidisciplinary tools and data types to hasten genetic gains and meet the rising food demand despite climate adversities. They combine genomic technologies with phenomics, transcriptomics, and metabolomics (multi-omics) integrated through AI-driven predictive models. These AI models facilitate the prediction of complex traits under variable environmental scenarios, enhancing precision in selection and breeding efficiency. By harnessing pangenomics, breeders capture the entire genomic variability within species, including rare and novel alleles for climate adaptation, overcoming limitations posed by single reference genomes. Together, these innovative strategies target not only crop yield but also sustainability traits such as resource-use efficiency and reduced greenhouse gas emissions. Such integrative breeding represents the frontier of developing climate-smart crops vital for future food security in an era of environmental unpredictability and change. To ensure climate-resilient genetic gain, breeding must adopt a standardized envirotyping pipeline, environmental covariate extraction, feature engineering, and reaction norm modelling in GS. This quantifies G×E interactions, providing a pointer to G×E modelling for predicting genotype performance across environmental gradients. Robust AI models require diverse Multi-Environment Trials (METs) spanning 10–20 site-years to capture stress variability. Prioritizing site-year diversity over replication prevents overfitting, ensuring varieties are climate-proofed. This framework transforms environment from noise into a selectable trait for resilient crop improvement.

## 7 Conclusion and future prospects

To enhance root, tuber, and cereal crop breeding through pangenomics, multi-omics integration, and AI-driven predictive models, we recommend addressing emerging issues centering on data complexity, model development, and practical application challenges. Implementing standardized frameworks to integrate diverse omics data–genomic, transcriptomic, proteomic, and metabolomic, will overcome technical obstacles from heterogeneous data formats and vast information scales. Prioritizing development of robust AI models capable of accurately predicting complex traits under varied environmental conditions, while incorporating explainable AI to address complex genotype-by-environment interactions and the need for interpretability in breeding decisions, is essential. Furthermore, establishing strong interdisciplinary collaborations between computational scientists, breeders, and field experts will enable translation of AI predictions and multi-omics insights into effective breeding strategies. Securing investment in access to high-quality phenotypic and environmental data, data standardization, and resource allocation for developing and deploying these technologies in breeding programs remains critical. Developing ethical governance frameworks around genetic data use and accessibility for smallholder farmers will address critical societal issues.

Despite these challenges, capitalizing on future prospects for applying pangenomics, multi-omics, and AI in climate-adaptive breeding is vital. Leveraging advancements in sequencing technologies and computational power to enhance the resolution and scale of pangenomes will capture more comprehensive allelic diversity crucial for adaptation traits. Deploying AI-driven models expected to become more sophisticated with improvements in deep learning and explainable AI will allow for better prediction and interpretation of genotype-phenotype relationships. Operationalizing integration of multi-omics data to enable breeders to dissect complex traits, accelerate gene discovery, and optimize genetic gain by precisely targeting stress tolerance mechanisms will accelerate progress.

Ultimately, focusing innovations on developing climate-smart crop varieties with improved yield stability, resource-use efficiency, and resilience to abiotic and biotic stresses should be prioritized. Implementing enhanced automation and digital breeding pipelines to reduce breeding timelines will facilitate rapid response to emerging climate challenges. The combined approach holds tremendous potential for addressing global food security and sustainability under ongoing climate change, particularly for key staple crops including roots, tubers, and cereals, with coordinated monitoring to track implementation progress.
