# Correction: Genetic enhancement of root, tuber and cereal crops via pangenomics, multi-omics integration and AI-driven prediction

**DOI:** 10.3389/fpls.2026.2016675

**Published:** 2026-10-12

**Authors:** Simbo Diakite, Prince E. Norman, Lansana Kamara, Necla Pehlivan, Meisam Zargar

**Affiliations:** 1 Department of Agrobiotechnology, Institute of Agriculture, Peoples Friendship University of Russia (RUDN) University, Moscow, Russia; 2 Germplasm Improvement and Seeds System, Sierra Leone Agricultural Research Institute (SLARI), Freetown, Sierra Leone; 3 Faculty of Art and Sciences, Department of Biology, Recep Tayyip Erdogan University, Rize, Türkiye; 4 Professor of plant protection, Department of Agrobiotechnology, Agrarian Technological Institute, RUDN University, Moscow, Russia

**Keywords:** artificial intelligence, crops, environmental adaptation, genetic enhancement, multi-omics, pangenomics, personalized breeding, precision agriculture

“Meisam Zargar” was misspelt as “Meisan Zargar” in the published article. This has been corrected.

An incorrect **Funding** statement was provided. The funder “Recep Tayyip Erdogan University Development Foundation, Grant Number 020260050120315” was erroneously omitted. The **Funding** statement has been corrected below.

“The author(s) declared that financial support was received for this work and/or its publication. This article was funded by Recep Tayyip Erdogan University Development Foundation, Grant Number 020260050120315.”

The original version of this article has been updated.

